# 5-ALA in Oncology: Current Clinical Applications, Biological Limitations, and Emerging Translational Strategies

**DOI:** 10.3390/biomedicines14061314

**Published:** 2026-06-10

**Authors:** Julia Inglot, Dorota Bartusik-Aebisher, Angelika Myśliwiec, Klaudia Dynarowicz, Avijit Paul, Marvin Xavierselvan, David Aebisher

**Affiliations:** 1 English Division Science Club, Faculty of Medicine, Collegium Medicum, University of Rzeszów, 35-310 Rzeszów, Poland; inglotjulia@gmail.com; 2 Department of Biochemistry and General Chemistry, Faculty of Medicine, Collegium Medicum, University of Rzeszów, 35-310 Rzeszów, Poland; dbartusikaebisher@ur.edu.pl (D.B.-A.); amysliwiec@ur.edu.pl (A.M.); kdynarowicz@ur.edu.pl (K.D.); 3 Department of Biomedical Engineering, Tufts University, Medford, MA 02155, USA; avijit.paul@tufts.edu (A.P.); marvin.xavierselvan@tufts.edu (M.X.); 4 Department of Photomedicine and Physical Chemistry, Medical College, University of Rzeszów, 35-310 Rzeszów, Poland

**Keywords:** 5-ALA, photodynamic therapy, photodynamic diagnostics, photosensitizer, oncology, cancers, reactive oxygen species, protoporphyrin IX, heme, fluorescence, oxygen

## Abstract

5-Aminolevulinic acid (5-ALA) has emerged as an important theranostic agent in oncology due to its selective intracellular conversion to protoporphyrin IX (PpIX), enabling both photodynamic diagnosis (PDD) and photodynamic therapy (PDT). This narrative review summarizes current knowledge regarding the biological mechanisms underlying 5-ALA metabolism, selective tumor accumulation, and the clinical applications of 5-ALA-based approaches across multiple oncological indications. Particular emphasis is placed on glioblastoma, head and neck lesions, dermatological malignancies, urological cancers, gynecological lesions, and emerging translational applications. The review also discusses key biological and technical limitations, including tumor hypoxia, restricted light penetration, heterogeneous PpIX accumulation, resistance mechanisms, and tumor-specific variability. Recent advances in drug delivery systems, nanotechnology, sonodynamic therapy, radiodynamic strategies, and combination immunotherapeutic approaches are also highlighted. Collectively, current evidence indicates that while 5-ALA has established clinical utility in selected indications, many applications remain preclinical or early translational, underscoring the need for further well-designed clinical studies.

## 1. Introduction

**Figure 1:**
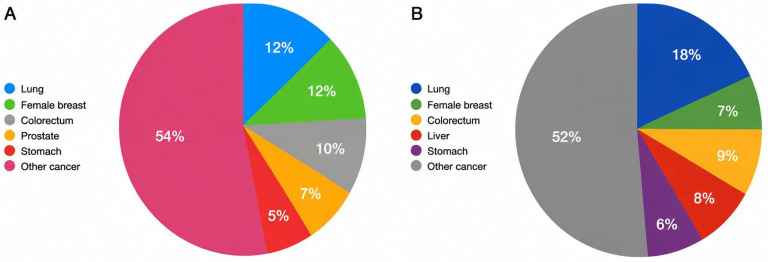
(**A**) Percentage share of the types of cancer in total new cancer diagnoses; (**B**) percentage share of the types of cancer in total cancer deaths.

According to statistics collected in the United States in 2024, cancer is the second most common cause of death overall, and the first in the group of people under 85 years of age. The COVID-19 pandemic had a negative impact on the diagnosis and treatment of oncological diseases, when access to specialists was difficult. It is estimated that the probability of invasive cancer diagnosis is 41.6% in men and 39.6% in women. It is noted that the number of new diagnoses in people over 65 years of age has decreased, in contrast to people under 65 years of age, where this number is increasing [1]. Figure 1 shows the percentage share of the most common types of cancer in total new cancer diagnoses in the world in 2022 [2].

Currently, the standard methods of treating cancer are surgery, chemotherapy and radiotherapy. Unfortunately, none of these methods is without its drawbacks; each has its side effects. Radical surgical treatment is not always possible. Examples of complications of radiotherapy are acute toxicity, vascular damage, and secondary tumors. Side effects of chemotherapy include inflammation of the gastrointestinal mucosa and, consequently, anorexia, fatigue or local pain [3]. Additionally, the treatment of metastases is often associated with resistance and the occurrence of changes in multiple locations [4]. Therefore, currently, emphasis is placed on the use of alternative treatment methods, with fewer side effects and selectivity for cancer cells. A promising solution is PDT [3].

The added value of this review lies in its integrative translational perspective. Rather than focusing on a single clinical indication or one technical aspect of 5-ALA/PpIX-based strategies, this analysis combines mechanistic, pharmacological, clinical, and emerging therapeutic perspectives. In particular, it discusses how tumor-specific heme metabolism, heterogeneous PpIX accumulation, hypoxia, transporter activity, resistance mechanisms, and tumor microenvironmental interactions influence the diagnostic and therapeutic performance of 5-ALA-based approaches. In addition, the review links established clinical applications with emerging optimization strategies, including 5-ALA derivatives, nanocarrier systems, sonodynamic and radiodynamic approaches, and immunotherapy-oriented combinations. Therefore, this review aims to provide a broader framework for understanding not only where 5-ALA/PpIX is clinically useful, but also why its efficacy varies across tumor types and how current translational barriers may be overcome.

## 2. Photodynamic Therapy and Photodynamic Diagnostics

The healing properties of light have been known since antiquity. Initially, it was credited with therapeutic effects for diseases such as skin cancer, psoriasis, and vitiligo, and later also in rheumatism, scurvy, rickets, paralysis, muscle weakness, edema, and tuberculosis. A significant event was the awarding of the Nobel Prize in Physiology or Medicine to Niels Finsen in 1903 for his achievements in this field. In 1907, the concept of “photodynamic action” was clarified, describing the reactions between light, oxygen, and certain substances [5]. In 1987, ALA-PDT was first used to treat both cancerous and non-cancerous skin conditions by the Kennedy and Pottier group, and in 1999, the FDA approved this method for the treatment of selected conditions [5,6].

The essence of PDT is the generation of reactive oxygen species in target tissue, most often cancer [7]. Photosensitizers (PSs) are activated by light of specific wavelengths corresponding to their absorption bands in the visible range—usually red or blue—as well as in the near-infrared region and even sunlight. Upon energy absorption, a PS transitions from its ground state to an excited triplet state [8]. This state is unstable and can return to the ground state via fluorescence emission or internal conversion [5].

In its excited state, PSs can participate in two types of reactions. In the type I reaction, direct interactions with biomolecules such as lipids, proteins, or amino acids occur, resulting in electron transfer and the formation of a superoxide anion radical (O_2_•−) and a HO_2_• radical. The superoxide anion radical then dismutates to hydrogen peroxide (H_2_O_2_), which can lead to the formation of the highly reactive hydroxyl radical (OH•). In the type II reaction, the energy of the excited PS is transferred directly to molecular oxygen, leading to the formation of singlet oxygen (^1^O_2_). Both mechanisms can occur in parallel, but in oxygen photodynamic therapy, the type II reaction predominates [8].

In addition to PDT, photosensitizers are also used in other therapeutic methods, such as sonodynamic therapy (SDT) using ultrasound, radiodynamic therapy (RDT) using ionizing radiation, microwave dynamic therapy (MDT), and electrodynamic therapy (EDT) using alternating current [7].

Besides its therapeutic applications, 5-ALA is also used in diagnostics [6]. This compound itself does not exhibit fluorescent properties, unlike its metabolite, PpIX [9,10]. After administration, 5-ALA is converted to PpIX, which emits red fluorescence under the influence of blue-violet light at a wavelength of 375–475 nm, enabling precise visualization of neoplastic lesions [6,11,12]. This method is used to detect precancerous lesions, early and primary tumors and metastases, for intraoperative tumor demarcation, and for monitoring the effects of PDT [4,13].

To date, PDD has been used primarily in the imaging of malignant gliomas, as well as in urology, gynecology, otolaryngology, gastroenterology, and pulmonology. The main limitations of this method include variable fluorescence intensity depending on the tumor type (e.g., weak fluorescence in gastrointestinal tumors, LGG, and HGG at the tumor borders), fluorescence in non-neoplastic lesions such as inflammatory foci, and background autofluorescence [6,9].

**Figure 2:**
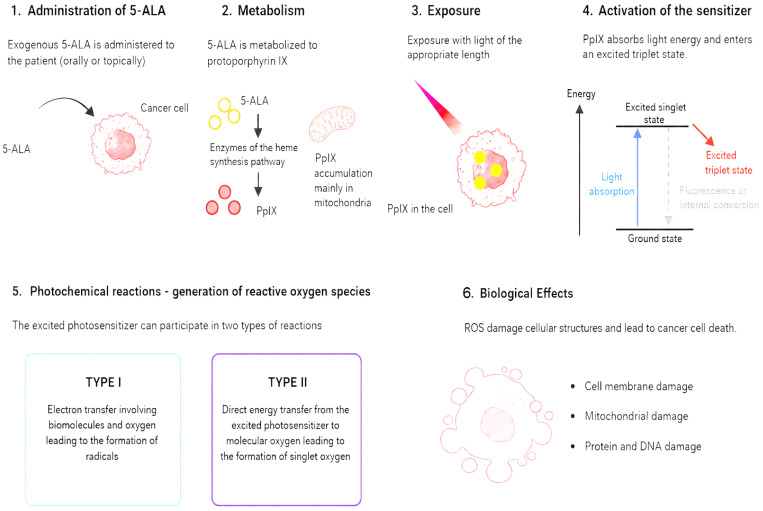
Schematic diagram of the mechanism of action of PDT using 5-ALA. After administration, 5-ALA is converted to PpIX, which accumulates in cancer cells. Irradiation with light of the appropriate wavelength leads to excitation of the photosensitizer and initiation of type I and II photochemical reactions, resulting in the generation of ROS, including singlet oxygen (^1^O_2_). The resulting ROS cause damage to cellular structures, leading to cancer cell death by apoptosis or necrosis.

The effectiveness of photodynamic therapy in oncology is described in detail in a review by Dolmans et al., who emphasize that PDT is a method for selectively destroying cancer cells by inducing oxidative stress and damaging oxygen-dependent cellular structures (Figure 2). The authors point out that tumor hypoxia remains a key limitation of the method, but they also emphasize its high selectivity and the possibility of repeated treatments without cumulative systemic toxicity [14].

A significant advancement in the clinical use of 5-ALA in photodynamic therapy for skin lesions was described by Kennedy and Pottier, who were the first to demonstrate the efficacy of exogenous 5-aminolevulinate in inducing the selective accumulation of protoporphyrin IX in diseased tissues. Their research formed the basis for the introduction of ALA-PDT in the treatment of superficial skin cancers and precancerous lesions, confirming the high efficacy of the therapy while limiting damage to healthy tissue [15].

In the field of intraoperative diagnosis of malignant gliomas, the studies by Stummer et al. were crucial. They demonstrated that the use of 5-ALA leads to the selective accumulation of PpIX in brain tumor cells, enabling their precise visualization during surgery under blue light. The authors proved that this method increases the rate of complete tumor resection and improves progression-free survival [16].

## 3. 5-Aminolevulinate (5-Aminolevulinic Acid) (5-ALA)

**Figure 3:**
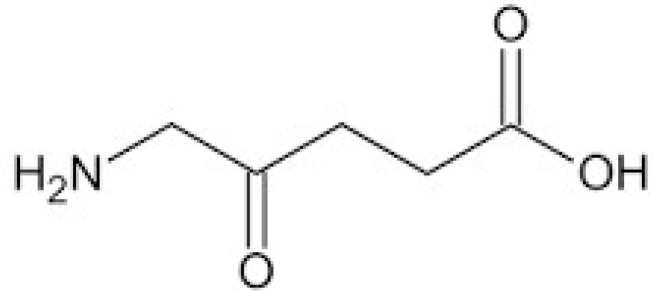
Chemical structures of 5-ALA.

5-ALA (Figure 3) is an amino acid produced naturally by animals and plants [17]. It is a prodrug, a hydrophilic precursor of chlorophyll and hemoglobin, necessary for the synthesis of heme—a protein regulating the formation of adenosine triphosphate in oxygen metabolism [3,9,17]. In the cell, it most likely accumulates in mitochondria [4].

### 3.1. Metabolism of 5-ALA in Cancer Tissues

**Figure 4:**
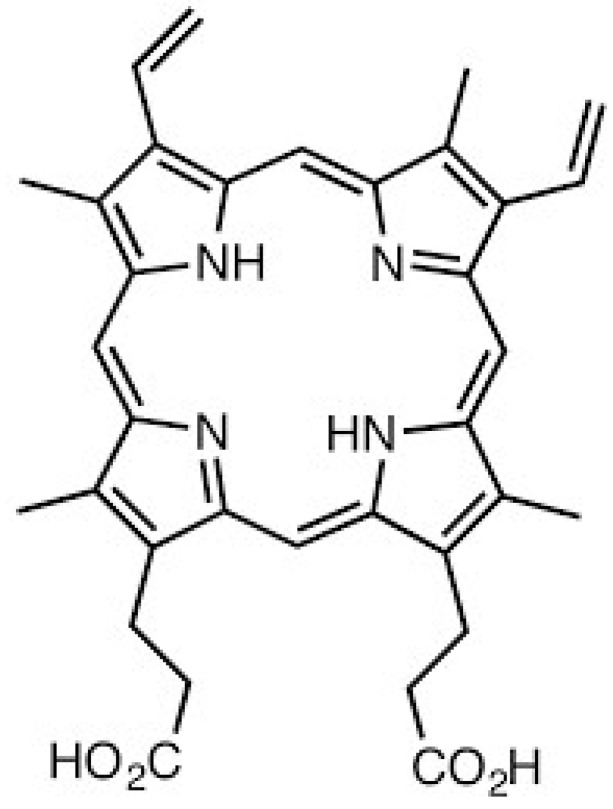
Chemical structure of protoporphyrin IX.

5-ALA can be exogenous and endogenous. Endogenous 5-ALA is produced in mitochondria with the participation of 5-ALA synthase (ALAS) from succinyl-CoA and glycine. Exogenous 5-ALA is delivered to the cell with the participation of peptide transporters (PEPT) 1 and 2. Condensation of two 5-ALA molecules with the participation of ALA dehydratase (ALAD) results in the formation of porphobilinogen (PBG) in the cytoplasm. With the participation of porphobilinogen deaminase (PBGD), hydroxymethylbilane (HMB) is obtained by condensation of four PBG molecules. From HMB in living cancer cells, uroporphyrinogen III (UPG III) is formed thanks to uroporphyrinogen III synthase (UROS). Then, by decarboxylation of all acetate groups, UPG III is transformed into coproporphyrinogen III (CPG III), which enters the mitochondria with the participation of the ATP-binding cassette transporter B6 (ABCB6). The next steps are the transformation of CPG III into protoporphyrinogen (Ppgen); Ppgen into PpIX, with the participation of ferrochelatase (FECH); and PpIX into heme, which regulates the activity of ALAS [9,18]. In healthy cells, exogenous 5-ALA is rapidly converted to heme. In cancer cells, alterations in the activity of enzymes involved in the heme biosynthesis pathway contribute to the accumulation of protoporphyrin IX (PpIX) (Figure 4).

In particular, decreased activity or expression of ferrochelatase (FECH), the enzyme responsible for incorporating ferrous ions (Fe^2+^) into PpIX to form heme, leads to reduced conversion of PpIX and its intracellular accumulation. Additionally, disturbances in iron homeostasis may further limit the availability of Fe^2+^ required for this reaction.

Moreover, cancer cells often exhibit increased expression of peptide transporters PEPT1 and PEPT2, which enhances the uptake of 5-ALA, leading to increased intracellular synthesis of PpIX. This accumulation is further associated with enhanced fluorescence intensity, improving the effectiveness of PDD [9,17] (Table 1).

**Figure 5:**
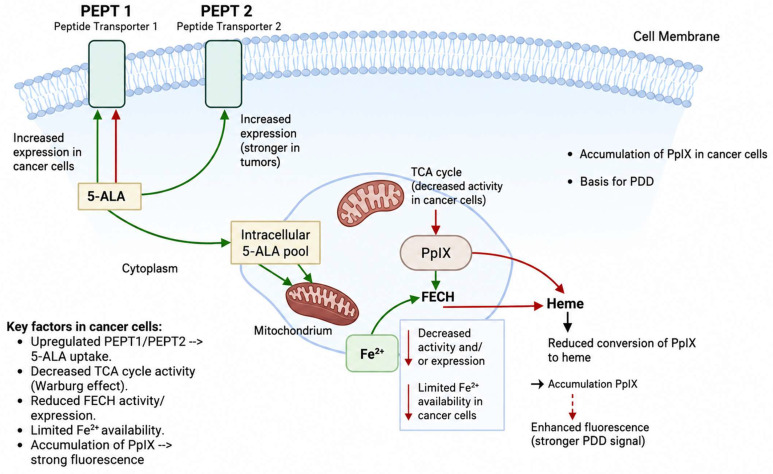
Schematic diagram of the metabolic pathways of 5-ALA in a living cancer cell. Increased expression of the peptide transporters PEPT1 and PEPT2 leads to increased 5-ALA uptake and increased intracellular concentration. Transformations in the heme biosynthetic pathway result in the formation of PpIX, which accumulates in cancer cells. This phenomenon is associated with reduced activity of the TCA cycle (Warburg effect), reduced availability of Fe^2+^ ions, and reduced activity or expression of FECH, responsible for the conversion of PpIX to heme. Consequently, heme synthesis is reduced and PpIX accumulates, resulting in increased fluorescence and providing the basis for PDD. Green arrows indicate active metabolic pathways and transport processes. Red arrows indicate reduced activity or impaired conversion pathways in cancer cells. Red dashed arrows indicate indirect downstream effects. Black arrows indicate biological consequences of the described mechanisms.

Studies indicate that PBGD and ALAD also show increased activity in cancer cells [6]. Studies have also shown that PpIX also accumulates in large amounts in pro-tumor components such as T lymphocytes, microglia and myeloid cells, which may increase the efficacy of treatment of, e.g., gliomas [7,19,20,21] (Figure 5).

PDT involves administering a photosensitizer and then irradiating the affected area with light of the appropriate wavelength, which leads to tissue damage [22]. In 5-ALA therapy, the photosensitizer is endogenously produced PpIX, which is formed following the administration of ALA and its transformation in the heme biosynthetic pathway [23]. ALA is metabolized in cells as part of the intrinsic heme synthesis pathway, leading to the accumulation of PpIX—a fluorescent compound that, when activated by light, generates reactive oxygen species, leading to cell death. This mechanism is used, among others, in the treatment of basal cell carcinoma [24,25]. It is also worth noting that this method has diagnostic applications, including the detection of bladder cancer, cervical intraepithelial lesions, and lung cancer and in fluorescence-assisted glioma surgery [26]. Under physiological conditions, 5-ALA is formed endogenously from succinyl-CoA and glycine via ALA synthase. It then undergoes a series of enzymatic reactions leading to the formation of PpIX and ultimately heme. This pathway is tightly regulated by iron availability and heme levels. Exogenous administration of 5-ALA (e.g., in the context of PDT) can lead to PpIX accumulation in cells as a result of increased 5-ALA concentration, increased ALA synthase activity, and dysfunction of FECH—the enzyme that catalyzes the incorporation of Fe^2+^ into PpIX and the formation of heme [27]. In cancers, reduced FECH activity has been demonstrated as one of the mechanisms promoting PpIX accumulation, explaining its preferential accumulation in neoplastic tissues. Reduced FECH expression has been observed in, among others, liver metastases of colon cancer, prostate cancer, bladder cancer, and colon cancer [28,29,30]. Another important enzyme is porphobilinogen deaminase (PBGD), which catalyzes one of the key steps in PpIX synthesis. High activity of PBGD, as well as ALA dehydratase and uroporphyrinogen decarboxylase, has been demonstrated in breast cancer cells, squamous cell carcinoma, and adenocarcinoma [31,32,33]. Another enzyme that influences PpIX fluorescence levels is coproporphyrinogen oxidase (CPOX), which catalyzes the formation of protoporphyrinogen IX from coproporphyrinogen III. CPOX expression correlates with PpIX fluorescence levels in glioma cells, and its activation can further increase its concentration [34]. It should be emphasized, however, that the mechanism of PpIX accumulation in cancer cells is more complex than a simple model based on reduced FECH activity and increased expression of efflux transporters such as ABCG2. Recent reports indicate that PpIX accumulation is not solely a cancer cell-autonomous process. According to the concept proposed by Adapa et al. (2024) [35], cancer-associated fibroblasts (CAFs) in the tumor microenvironment can actively produce and release porphyrins, leading to a phenomenon termed “porphyrin overload”. This suggests a significant role for interactions between cancer cells and the tumor stroma in regulating PpIX levels. This stromal component further supports the notion that porphyrin homeostasis within tumors is the result of a dynamic exchange between cancer cells and their microenvironment, rather than solely a consequence of intracellular enzymatic regulation. In this view, CAF-derived porphyrins may constitute an additional source of energy for the heme biosynthetic pathway in cancer cells, leading to a further increase in intracellular PpIX accumulation beyond that resulting solely from intrinsic tumor cell perturbations. Such intercellular metabolic cooperation may also explain the discrepancies previously observed in the correlations between classical pathway components and PpIX levels across different cancer types and stages [36,37,38]. Furthermore, this model fits well with the concept of a metabolically active tumor niche, in which both cancer cells and stromal elements contribute to the formation of a common pool of porphyrins. The resulting state of “porphyrin overload” may increase the susceptibility of tumor tissue to redox imbalance and oxidative stress, amplifying the previously described sensitivity resulting from endogenous porphyrin production and their interactions with therapeutic modulators [39,40,41]. Consequently, targeting therapy to porphyrin metabolism may require a broader perspective encompassing not only intracellular pathways of tumor cells but also cooperative metabolic exchanges occurring within the tumor stroma [42,43]. The role of efflux transporters, such as ABCG2, also appears to be context-dependent. Studies conducted on a panel of JFCR39 cell lines demonstrated a weak correlation between ABCG2 expression and PpIX shedding in multiple cancer types. This suggests the involvement of alternative mechanisms, such as dynamin-2-dependent exocytosis, which may play an important role in regulating porphyrin levels in cells [44]. 5-ALA uptake, in turn, is strongly dependent on the peptide transporters PEPT1 and PEPT2, whose expression is often increased in cancer cells. PEPT2 plays a particularly important role in the central nervous system. Studies using PEPT2 knockout models have shown an approximately 62% reduction in ALA uptake by astrocytes, indicating its crucial role in the mechanism of fluorescence used during glioma surgery. Furthermore, accumulating evidence indicates significant heterogeneity in PpIX accumulation between different subpopulations of cancer cells. Cancer stem cells (CSCs) exhibit inherent resistance to 5-ALA-PDT, which is associated with lower levels of PpIX and increased expression of ABC family transporters. Conversely, dormant cancer cells may accumulate higher amounts of PpIX, which is associated with increased expression of PEPT1 and ABCB6 and decreased expression of ABCG2 [45]. Mechanisms of acquired resistance to PDT that extend beyond efflux transporters are also important. It has been shown that repeated 5-ALA-PDT therapy, even when combined with ABCG2 inhibition, can lead to the development of resistance by reducing the levels of heme biosynthetic enzymes. In particular, a decrease in PBGD levels has been identified as a resistance mechanism in glioma cells, suggesting the need to monitor not only the transporters but also the enzymes of the heme biosynthetic pathway [46]. The initial stages of 5-ALA metabolism are analogous to those in living tumor tissue. In necrotic cells, due to the lack of uroporphyrinogen III synthase (UROS) activity, non-enzymatic formation of uroporphyrinogen I (UPGI) occurs instead of uroporphyrinogen III, which is characteristic of 5-ALA metabolism in dead tumor cells [9].

### 3.2. Features of 5-ALA

5-ALA during PDT can be administered orally, topically or intravenously [4]. Orally administered 5-ALA is characterized by low bioavailability [3,47]. However, the half-life of skin photosensitization it causes is short, which gives it an advantage over other PSs [6]. When applied topically, it does not cause generalized photosensitivity, which is why it is widely used in the treatment of dermatological conditions [3,47] (Table 2). PpIX is activated using light with a wavelength in the range of 580 nm to 740 nm, optimally 635 nm, which can come from various sources, such as lasers, xenon lamps, mercury lamps, halogen lamps or LED light [3,10,48].

After systemic administration, the 5-ALA metabolite, PpIX, accumulates mainly in the tissues forming the surface and in the glands with ducts opening onto the former, i.e., the urothelium, endometrium, epidermis, skin, oral mucosa, gallbladder, or bile ducts. The level of PpIX fluorescence after oral administration of 5-ALA in the skin reaches a maximum in the range of 6.5 to 9.8 h, depending on the body area. In plasma, maximum values were observed 6.7 h after oral administration, 4.1 h after inhalation and 2.9 h after intravesical administration; in the case of local application of ALA, PpIX was not detectable. Oral administration of 5-ALA has an advantage over the intravenous route, as it is characterized by fewer side effects—it can cause mild gastrointestinal disorders and a decrease in blood pressure, pulmonary artery pressure and vascular resistance in the pulmonary circulation [3]. One of its main advantages is that 80% of this substance is eliminated from the body within 24 h, resulting in no photosensitivity reactions. Furthermore, its oral bioavailability is good; the substance should be administered to the patient 2–4 h before the planned procedure [21,49]. 5-ALA is characterized by a small volume of distribution after systemic administration, which indicates its poor pharmacokinetic properties—a significant part of the drug is excreted in urine in an unchanged form or metabolized in the liver. For this reason, esterified ALA derivatives have been developed, which offer numerous advantages, such as better pharmacokinetic properties, greater stability, lower doses, deeper penetration into tissues, more uniform and higher PpIX concentration and shorter application time [6].

Contemporary literature reviews confirm that 5-ALA is a prodrug widely used in both fluorescence diagnostics and photodynamic therapy, and its action is based on the endogenous synthesis of PpIX in the heme pathway. After systemic administration, 5-ALA undergoes intracellular conversion to PpIX, which exhibits photosensitizing and fluorescent properties, which forms the basis of its clinical applications in brain tumors, the bladder, and skin surgery [50,51]. At the same time, it is emphasized that the selective accumulation of PpIX in tumor tissue results from a combination of heme pathway enzyme activity and differences in cellular metabolism, which remains a key element of the effectiveness of this therapeutic strategy [51].

The pharmacokinetics of 5-ALA have been intensively studied in recent studies, which point to its limitations, such as low lipophilicity, variable bioavailability, and the dependence of the effect on the route of administration and exposure time. After oral administration, PpIX is observed to rapidly appear in tissues, with maximum fluorescence usually occurring within a few hours, which is crucial for optimizing PDT and fluorescent surgery procedures [50,52]. The literature also points out that parameters such as light wavelength (~635 nm), fluence, and the time from 5-ALA administration to irradiation have a direct impact on the efficiency of reactive oxygen species generation and the effectiveness of tumor cell destruction [53].

In response to the limitations of classical 5-ALA, its ester derivatives and nanocarrier systems are being developed to improve stability, bioavailability, and tissue penetration. New prodrug forms enable more uniform and higher concentrations of PpIX in tumor tissues, which increases the efficacy of both fluorescence diagnostics and photodynamic therapy [54,55]. The development of these strategies indicates that the future applications of 5-ALA will be related not only to optimizing the molecule itself but also to controlling its distribution in the tumor microenvironment and improving the selectivity of PpIX accumulation.

### 3.3. Modifications

Attempts are being made to modify the 5-ALA molecule to increase the effectiveness of 5-ALA-PDT. An example is hexyl ester-5-ALA, which, through extension of its side chain, achieves better cellular penetration and lipid solubility. Its efficacy in PDT has been demonstrated in the treatment of drug-resistant human uterine sarcoma. Furthermore, compared to ALA, it has a stronger photosensitizing effect [56,57]. Another modification resulted in the hexenyl ester-ALA, which has been shown to be effective as a PS in PDT in the treatment of human breast cancer, particularly adriamycin-resistant MCF-7 [48,58]. Teper et al. demonstrated that an esterified 5-ALA derivative with longer lipophilic side chains may be helpful in the treatment of castration-resistant prostate cancer [59]. Another modification is AlaAcBu, a prodrug of ALA that, via tumor esterase, causes the release of butyric acid, acetaldehyde, and ALA. Studies show that this substance is more effective than ALA and may be effective in the treatment of doxorubicin-resistant breast cancer [56,60]. Also of hope are the Schiff base derivative N-3′,5′-dichloro-2′-hydroxybenzylidene-2-chloro-4-nitroaniline, or TX-816, which may prove effective in the fight against resistant cancer cells, as well as methyl-ALA (Me-ALA) and hexyl-ALA, which accumulate more selectively in the tumor [25,56,61].

Current research indicates that the effectiveness of 5-ALA derivatives in photodynamic therapy depends not only on their transport properties but also on their ability to bypass early metabolic limitations in the cell and modulate the rate of entry into the heme biosynthetic pathway. It has been shown that 5-ALA prodrugs with modified chemical structures can increase the pool of available substrates for PpIX synthesis through more efficient release of ALA in the intracellular environment, which translates into a higher photodynamic effect even at lower starting doses of the compound. More recent analyses also emphasize that the limitations of classic 5-ALA lie in its instability and rapid metabolic processing, which has become a key premise for the design of more stable prodrugs and systems activated in the tumor microenvironment [50,62].

**Figure 6:**
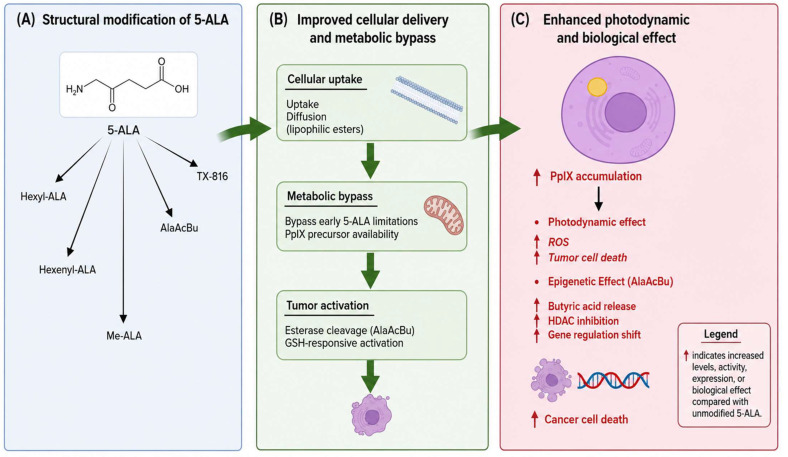
Conceptual overview of 5-ALA optimization strategies. (**A**) Conceptual representation of major optimization strategies applied to 5-ALA-based PDT. (**B**) Improved pharmacokinetic and intracellular properties of 5-ALA derivatives, including enhanced cellular uptake, enhanced diffusion across biological membranes, and partial bypass of early metabolic constraints, leading to greater availability of precursors for PpIX synthesis. Selected prodrugs are activated in the tumor microenvironment through enzymatic cleavage, such as by esterases, or glutathione (GSH)-responsive mechanisms. (**C**) Improved therapeutic outcomes, including increased intracellular PpIX accumulation, increased ROS production upon photoactivation, and improved tumor cell killing. Furthermore, selected derivatives, such as AlaAcBu, may exert epigenetic effects through the release of butyric acid, resulting in histone deacetylase (HDAC) inhibition and modulation of gene expression. These combined mechanisms may contribute to improved efficacy of PDT, particularly in drug-resistant tumor models.

Simultaneously, multifunctional 5-ALA conjugate strategies are being developed that combine the classic phototoxicity of PpIX with additional biological mechanisms that influence cancer cell survival. For example, some AlaAcBu-type prodrugs simultaneously release metabolites with epigenetic activity, such as butyric acid, which can modulate the expression of genes involved in proliferation and oxidative stress response by inhibiting histone deacetylases (HDACs) (Figure 6). This approach leads to a synergistic enhancement of the PDT effect by simultaneously disrupting redox homeostasis and epigenetic regulation of cancer cells. This concept fits into the broader trend of designing “hybrid photodynamic prodrugs” that not only generate PpIX but also actively modify the tumor cellular microenvironment [62,63].

## 4. Pharmacokinetics and Formulation Advances

5-ALA formulations used in the treatment of gliomas focus on overcoming key limitations such as the blood–brain barrier (BBB) and tumor heterogeneity, often utilizing hydrogels, liposomes, nanoparticles, and long-acting depot systems that enable local and sustained drug release. These advanced delivery systems aim to increase drug retention in tumor tissue, reduce systemic toxicity, and improve therapeutic efficacy through controlled release at the tumor site. Examples include hyaluronic acid-based hydrogels for targeted drug delivery and nanodiscs designed to cross the BBB and transport combined therapies [64].

Biocompatible polymer matrices play a particular role in these systems, enabling time- or stimulus-dependent (e.g., temperature-dependent) release, allowing for precise control of tumor exposure to 5-ALA and its metabolites [65]. 5-ALA remains a key compound used in the diagnosis and treatment of brain tumors, particularly in the fluorescence-assisted resection procedure for gliomas (Gliolan), where selective accumulation of PpIX enables visualization of tumor tissue during surgery and increases the rate of tumor resection [66].

Current nanotechnological approaches focus on improving the bioavailability of 5-ALA through its encapsulation in nanocarriers such as PEG-chitosan, HPMA, or liposomes. Nanoparticles have been shown to increase 5-ALA stability, improve its accumulation in tumor cells, and intensify the photodynamic effect after irradiation, leading to stronger ROS generation and increased cytotoxicity [67,68]. This mechanism also relies on the EPR (enhanced permeability and retention) effect, which enables passive accumulation of nanocarriers in tumor tissue.

More recent studies emphasize that nanocarriers can simultaneously modulate the tumor microenvironment by improving oxygen availability and reducing hypoxia, which directly increases the effectiveness of PDT. For example, platforms based on gold nanoparticles (AuNPs, AuNRs) can enhance singlet oxygen generation through plasmonic effects, increasing the efficiency of photochemical reactions [69]. Chitosan-based systems, in turn, enable the simultaneous delivery of 5-ALA and other therapies, such as gene therapies, paving the way for combination therapies [67].

Another important area of development is intelligent nanocarriers with a hollow core structure, which enable the rapid release of 5-ALA in the tumor environment, increasing the local concentration of the prodrug and the effectiveness of PDT [55,70,71]. Recent reviews indicate that such systems can be functionalized with targeting ligands or equipped with controlled release mechanisms dependent on the tumor microenvironment, which increases selectivity towards cancer cells and reduces toxicity in healthy tissues [72,73].

Additionally, recent work highlights the development of multifunctional nanotherapeutic platforms that combine 5-ALA with photothermal therapy (PTT), gene therapy, and hypoxic oxygen-generating systems. This approach overcomes one of the main limitations of PDT—tumor hypoxia—and significantly increases treatment efficacy in glioma and solid tumor models [55,72].

Advanced 5-ALA delivery systems with the greatest translational potential currently include liposomal formulations, polymer-based nanoparticles, PEG/chitosan-based nanocarriers, hollow mesoporous silica nanoparticles, hydrogel or depot systems for sustained local release, and multifunctional oxygen-modulating nanoplatforms. These systems may improve 5-ALA stability, increase tumor-selective accumulation, prolong local exposure, enhance intracellular PpIX generation, and reduce systemic toxicity. Local delivery platforms, such as hydrogels or depot systems, appear particularly promising for surgically accessible tumors, including glioblastoma, where administration directly into the resection cavity could increase local drug retention and reduce off-target exposure. Stimuli-responsive nanocarriers activated by pH, redox status, hypoxia, or tumor-associated enzymes may further improve selectivity and enable more controlled 5-ALA release within the tumor microenvironment. Nevertheless, the main limitations of these approaches include limited clinical validation, potential nanomaterial-related toxicity, variable biodistribution, manufacturing complexity, regulatory challenges, and uncertain reproducibility between preclinical models and human tumors. Therefore, although advanced delivery systems represent one of the most promising directions for improving 5-ALA-PDT, their clinical translation will require standardized pharmacokinetic, safety, and efficacy studies [54,55,71,74,75].

## 5. Clinical Applications and Limitations

PDT can be successfully used in oncological treatment, especially as adjuvant therapy. Moreover, tissue sensitivity to PDT is not affected by previously used chemotherapy or radiotherapy and no tumor resistance is observed [3,4].

### 5.1. Central Nervous System Tumors

Glioblastoma multiforme, or grade IV glioma, is the most common and aggressive malignant brain tumor [7,76]. In adults, it accounts for 50% of gliomas [76]. It is estimated that 4–6 in 100,000 people are diagnosed with this disease annually, with men being diagnosed about 1.6 times more often than women [11,77]. Standard treatment for glioblastoma multiforme includes surgery combined with chemotherapy and radiotherapy, but each of these methods carries numerous side effects [7]. The median survival time is 15 to 30 months, and only 2% to 10% of patients survive 5 years [7,76,77]. The number of relapses is very high and reaches up to 90%, with 80% of relapses located in the area of the resection cavity, which would suggest that the resection margin was insufficient [76,77].

In vitro studies have demonstrated the high efficacy of 5-ALA in selectively accumulating PpIX in glioma cells. In GIC7 and PG88 models, Pedrosa et al. demonstrated that PpIX is detectable approximately 1 h after exposure, and its fluorescence emission increases and stabilizes within approximately 24 h. Irradiation with 635 nm light led to a significant increase in apoptosis and inhibition of cancer cell proliferation, while simultaneously demonstrating no significant phototoxicity to healthy cells. The cytotoxic effect was dependent on both the 5-ALA concentration (up to 50 µg/mL) and the light dose, achieving almost complete cell elimination at higher exposure parameters [77].

In vivo, Tétard et al. demonstrated the efficacy of interstitial photodynamic therapy with 5-ALA in a U87 rat glioma model. The use of 5-ALA at a dose of 100 mg/kg and irradiation with a 635 nm diode laser led to a pronounced cytotoxic effect, which was strongly dependent on the fluence parameters and the method of light exposure. High energy delivery rates and fractionated irradiation were observed to result in a higher rate of tumor necrosis, while lower fluences were primarily associated with a limited necrotic effect and better tissue tolerance. At the same time, no significant phototoxicity was observed in distant areas of healthy tissue, although local peritumoral edema was observed [78].

Clinical data support the use of intraoperative 5-ALA-based PDT as an adjunct to glioma surgery. In a study by Vermandel et al., in patients with GBM, oral administration of 5-ALA (20 mg/kg) before surgery and intraoperative irradiation with a dose of 200 J/cm^2^ led to promising survival outcomes, with a median survival of 23.1 months and a 1-year survival rate of 60%, while there were no serious adverse events associated with PDT [79]. These data support the potential for integrating PDT with surgical treatment and standard adjuvant therapy.

However, the significant diagnostic and therapeutic limitations of 5-ALA in lower-grade gliomas should be emphasized. Visible fluorescence occurs in only 5–52% of patients, while quantitative techniques such as PpIX spectroscopy can detect porphyrins in an additional 40–50% of cases that are not macroscopically visible. Antiepileptic drug therapy is also a significant confounding factor, significantly reducing fluorescence intensity (73% of treated patients vs. 17% without antiepileptic drug therapy; *p* = 0.046), which should be taken into account when interpreting intraoperative results.

Additionally, recent clinical studies describe the development of randomized phase III trials evaluating the role of 5-ALA in the treatment of glioma. The RESECT trial (2023) demonstrated a significantly higher rate of complete macroscopic resection with 5-ALA (79.1% vs. 47.8%; *p* = 0.0002), confirming its importance as a standard in glioma surgery. Furthermore, long-term follow-up of the INDYGO trial (2024) suggests a potential improvement in overall survival, particularly when combined with PDT, with further analyses underway in the DOSINDYGO trial (NCT04391062). Early-phase clinical trials, such as Pentalafen/Heliance (NCT05736406), are also currently underway, evaluating intraoperative PDT with 5-ALA in a clinical setting in the USA.

Primary brain tumors also include medulloblastoma, typically located in the cerebellum and fourth ventricle. They most often affect children aged 1–9 years, and metastases are diagnosed in up to 1/3 of them at the time of diagnosis. Similarly to glioblastoma multiforme, complete surgical resection is the basis of treatment. Briel-Pompka et al. conducted a study on MB Med8A, UW228-2, and ONS76 cell lines, which they exposed to 5-ALA-PDT and 635 nm light. The total delivered radiation dose was 18.75 J/cm^2^. PpIX accumulation was significantly higher after longer incubation times (min. 4 h) and at higher 5-ALA concentrations (100 μg/mL) compared to 2 h incubation time and a 5-ALA concentration of 12.5/25 μg/mL. The percentage of apoptotic cells increased with 5-ALA concentration [80]. Another tumor of the central nervous system is meningioma. It is estimated that it affects 2 to 7 per 100 thousand women and 1 to 5 per 100 thousand men per year. The basis of treatment is surgical resection and the recurrence rate reaches 10 to 20%. El-Khatib et al. exposed primary cultures of meningioma cells to 5-ALA-PDT, assessing their susceptibility. Cell viability significantly decreased with increasing 5-ALA concentration from 96.5% ± 7.6% at 12.5 μg/mL to 13.8% ± 7.5% at 100 μg/mL [81].

### 5.2. Head and Neck Lesions

Head and neck cancers include mucosal lesions arising in the oral cavity, lips, salivary glands, nasal cavity, paranasal sinuses, pharynx and larynx. Histologically, over 90% of them are squamous cell carcinomas. The 5-year survival is <50%, and approximately 650,000 people worldwide are diagnosed with this disease annually. Local recurrence is common, while metastases are relatively rare. The main factors contributing to pathogenesis are HPV infection, alcohol consumption, tobacco smoking and betel chewing [82]. Liu et al. conducted a cohort study of 75 patients to evaluate the efficacy of 5-ALA-PDT in the treatment of potentially malignant oral lesions, such as oral erythroplakia, oral leukoplakia, oral submucous fibrosis, oral lichen planus, oral lichenoid lesions, and chronic discoid lupus erythematosus. A positive effect of therapy was observed on 92% of patients, including 38.7% of patients achieving complete remission and 53.3% achieving partial response [83]. In their phase I study, Ahn et al. used 5-ALA-PDT in the treatment of premalignant and early superficial head and neck lesions. After 3 months of treatment, complete response was achieved in 69% of patients, with 5-ALA concentrations for 50 J/cm^2^ being 71%, 100 J/cm^2^—50%, 150 J/cm^2^—75% and 200 J/cm^2^—75%. Local recurrence for these 5-ALA doses was 57%, 33%, 25% and 25%, respectively [84]. After 24 months of follow-up, local control was observed in 57.7% of patients [85].

### 5.3. Breast Cancer

Many therapeutic methods are currently used to treat breast cancer, including surgery, hormone therapy, chemotherapy, radiotherapy, and HER2-targeted therapy [86,87]. It is estimated that in the United States alone, more than 300,000 cases of breast cancer will be diagnosed in 2024, and more than 40,000 patients will die from it [86]. Based on histological and molecular characteristics, breast cancers are divided into tumors expressing estrogen and/or progesterone receptors, HER2-positive tumors, and triple-negative breast cancer (TNBC), which does not express ER, PR, or HER2 [87].

It should be emphasized that most of the available data on PDT with 5-ALA in breast cancer are preclinical and in vitro, and there is currently no clear clinical evidence for this indication. Eskiller et al. conducted studies on two human breast cancer cell lines: MCF-7 (ER/PR-positive, HER2-negative) and MDA-MB-231 (TNBC). They demonstrated a 5-ALA concentration- and incubation time-dependent increase in PpIX accumulation, with significantly higher fluorescence observed in MDA-MB-231 cells compared to MCF-7 [88].

The higher sensitivity of TNBC cells was also evident in cell survival assays following irradiation. The strongest photodynamic effect was observed at a 5-ALA concentration of 1 mM and radiation doses of 9–12 J/cm^2^, compared to those at higher 5-ALA concentrations at lower energy doses [88]. Similarly, Kamanli et al. demonstrated that higher doses of light energy (18–30 J/cm^2^) led to an increased number of apoptotic cells, with this effect being more pronounced in the MDA-MB-231 cell line, and optimal efficacy was achieved at a dose of 18 J/cm^2^ [89].

The observed greater sensitivity of TNBC cells compared to the MCF-7 cell line may result from molecular differences between breast cancer subtypes, including differential expression of ABC family membrane transporters, such as ABCG2, which participate in the transport and efflux of porphyrins, including PpIX. This may partially explain the differences in PpIX accumulation and photodynamic efficacy between the studied cell lines. It should be emphasized that despite promising preclinical results, their translation into the clinical setting remains limited and requires further study.

### 5.4. Gynecological Malignancies and Premalignant Lesions

The number of gynecological cancers is on the rise, due to changes in lifestyle, genetic factors, and dietary habits. This group of cancers includes cancer of the uterine body, cervix, ovary, fallopian tube, vagina, and vulva, of which the first three are the most common. They constitute 1/3 of all cancers diagnosed in women [90]. It is estimated that in 2020, cervical cancer was the cause of cancer-related deaths in every fourth woman in the world [88]. Currently used therapeutic methods include surgery, chemotherapy, radiotherapy, targeted therapy, and immunotherapy [91].

High-grade squamous intraepithelial lesions (HSILs) are precancerous changes of cervical cancer. In turn, the main factor leading to this condition is considered to be infection with human papillomavirus (HPV). In Hu et al.’s study, they proved that topical 20% 5-ALA-PDT and 100 J/cm^2^ radiation in patients with HSIL and high risk of HPV can be an effective therapeutic option. The overall percentage of HSIL regression in the 22 patients was 90.91% after 6 months of follow-up, and the overall HPV clearance rate after this time was 86.36% [92]. In He et al.’s study, the cervical cancer cell lines SiHa, HT3, C4I, Caski, HeLa, C-33A, and Mel80 were treated with 5-ALA-PDT and their sensitivity was assessed. Cell proliferation was inhibited the most in the case of the Mel80 cell line, and the inhibition effect was directly proportional to the concentration of 5-ALA used (from 0.0001 to 0.1 mmol/L); with higher concentrations of 5-ALA (from 0.1 to 10 mmol/L), a plateau was achieved. The greatest decrease in cell viability was achieved after an incubation time of 4 h, gradually decreasing after 6, 12 and 24 h. The effects were similar regardless of the laser energy used—10 J, 20 J or 30 J [93]. HPV infections, apart from cervical intraepithelial neoplasia (CIN), may cause vaginal intraepithelial neoplasia (VaIN), which is also a precancerous condition. Han et al. conducted a retrospective study on a group of 303 patients with CIN or VaIN using 5-ALA-PDT, where a single dose of 5-ALA was 38 mg/cm^2^, the number of cycles ranged from 1 to 3, and the light density was 80 mW/cm^2^. After 6 months of treatment, the remission rates were: CIN 1—93.1%, CIN 2—90.6%, CIN 3—88.5%, VaIN 2—87.3%, and VaIN 3—77.8%. After 6 months, the HPV clearance rate was 72.5% [94]. Zhao et al. proved that the combination of superficial shaving with 5-ALA-PDT using a laser with a power of 100 mW/cm^2^ in the treatment of patients with a premalignant vulvar intraepithelial neoplasia lesion brings promising results. After 12 months, the clinical response among 17 patients was 94%, and very good cosmetic results were observed in 71% of patients [95]. Teshigawara et al. conducted a study on seven human clear cell ovarian cancer cell lines, of which OVMANA, RMG1, and RMG2 were found to be sensitive to 5-ALA-PDT in a dose-dependent manner. The 5-ALA concentration ranged from 0 to 1000 μM, and the power density was 17.4 mW/cm^2^. The ES2 line was resistant, while OVTOKO, KOC7C, and TOV21G were classified as intermediate sensitive. Half maximal inhibitory concentration (IC50) values were as follows: ES2, 882 μM; TOV21G, 330 μM; KOC7C, 857 μM; OVTOKO, 244 μM; RMG1, 56 μM; RMG2, 56 μM; and OVMANA, 97 μM [96].

### 5.5. Urological Cancers

Urological cancers mainly include prostate adenocarcinoma and upper urinary tract cancer, including bladder cancer, kidney cancer, penile cancer, and testicular cancer [97]. According to statistics collected in 2022 in the United States, prostate cancer accounted for 27% of all new cases of cancer diagnosed in men, bladder cancer for 6%, and kidney and renal pelvis cancer for 5%. Prostate cancer accounted for 11% of all cancer deaths in men, and bladder cancer accounted for 4% [98].

Kreigmair et al. conducted a study aimed at demonstrating the efficacy of 5-ALA-PDT in local therapy for superficial bladder cancer. A group of 10 patients were given intravesical enemas with 5 g of 5-ALA. The inside of the bladder was then exposed to light of 635 nm or 514 nm at a dose of 15, 30, or 60 J/cm^2^. After 10 to 12 weeks, 40% had complete remission, 20% had partial remission, 30% had no change, and 10% had progression. No serious side effects were observed [99]. Waidelich et al. reported the results of a study conducted on four patients with papillary lesions of the upper urinary tract after oral administration of 40 mg/kg of 5-ALA. The light used was 514 nm or 635 nm. Complete remission was observed in 50% of the patients, which was maintained at 7 and 17 months of follow-up. The other half of the patients had small residual tumors that were easily removed by a laser at follow-up [100]. Panetta et al. used 5-ALA-PDT to treat prostate cancer in vivo, in combination with 15-MV radiation and carbamide peroxide, creating radiodynamic therapy. Male mice were injected with PC-3 cells—human prostate cancer cells—and exposed to 15-MV, carbamide peroxide and 5-ALA in various combinations. Each of the methods used alone only slightly delayed tumor growth. The application of radiodynamic therapy resulted in inhibition of tumor growth after 1 week of treatment by 24 ± 9% and after 2 weeks of treatment by 21 ± 8% compared to radiotherapy. Overall, they contributed to slowing tumor growth relative to the control group by 39.4 ± 4.9% after 2 weeks of treatment [101].

### 5.6. Gastrointestinal Cancers

Gastrointestinal cancers are among the most frequently diagnosed and fatal cancers. The most frequently diagnosed of them in 2020 was colon cancer (colon cancer and rectal cancer). Second place was occupied by stomach cancer, followed by pancreatic, liver, esophageal and bile duct cancers. The highest number of deaths in 2020 among digestive system cancers was due to colon cancer, stomach cancer, pancreatic, liver, bile duct and esophageal cancer [102].

In the context of Barrett’s esophagus with high-grade dysplasia (HGD), it is important to emphasize that radiofrequency ablation (RFA) has largely replaced PDT as the first-line treatment. PDT, including 5-ALA, currently has primarily historical significance or as an alternative option in selected cases, but is not a standard treatment for HGD.

Older clinical trials have demonstrated high efficacy of 5-ALA-PDT, reaching 77–100% complete response in eradicating precancerous lesions. Kohoutova et al., in a study of 64 patients with Barrett’s esophagus, compared the efficacy of 5-ALA-PDT with PDT using photophorin. In the 5-ALA group, complete regression of intestinal metaplasia was achieved in 55% of patients compared to 22% in the Photofrin-PDT group, while regression of dysplasia was observed in 65% and 48% of patients, respectively [103].

However, long-term follow-up over five years has shown that the differences between 5-ALA-PDT and Photofrin-PDT are diminishing, and the final therapeutic effects of both methods are becoming comparable. These data should now be interpreted in the context of the development of more effective and less invasive ablative methods, such as RFA, which have led to a change in the standard of care for this group of patients.

Hino et al. performed 5-ALA-PDT on MKN-45, MKN-74 and NUGC-4 cell lines. In each of the three cell lines irradiated with a diode with a power of 3.0 J/cm^2^, a decrease in cell viability was observed, more under the influence of violet light than green or red light [104]. Hatakeyama et al., using the HT-29 colon cancer cell line, showed that under the influence of 5-ALA-PDT, cell viability was inhibited, and this value was proportional to the fluence (1.5 J, 3 J, 6 J). Additionally, in a study on mice with implanted HT-29 cells, under the influence of various LEDs, tumor growth was inhibited by up to 88% compared to the control group [105]. In studies on three hepatocellular carcinoma cell lines, HuH7, Hep3B and HepG2, in a mouse model of hepatocellular carcinoma and on primary samples of hepatocellular carcinoma patients, Kumar et al. demonstrated a positive effect of 5-ALA-PDT on the increase in the number of apoptotic cells. In each of the study groups, a significant decrease in cell proliferation was observed compared to the control groups [106]. Ozten et al. determined the effect of this therapy in vitro on hepatocellular carcinoma cell lines Huh-7 and SNU-449 (infected with HBV). Exposure of cells to light at energies of 3, 6, 9 and 12 J/cm^2^ resulted in a significant inhibition of cancer cell proliferation. The level of intracellular PpIX and the cytotoxic effect were more pronounced in the SNU-449 cell line. In the SNU-449 cell line, the highest percentage of apoptotic death and decrease in cell viability was achieved at an energy of 12 J/cm^2^, and in the case of the Huh-7 line, at an energy of 9 J/cm^2^ [107].

Regula et al., in their study using a model of pancreatic cancer in golden Syrian hamsters, showed the occurrence of necrosis from the tumor borders under the influence of 5-ALA-PDT, the extent of which increased over time. The applied dose of 5-ALA was 400 mg/kg, and the irradiation dose was 50 J/cm^2^. In the control group not exposed to PDT, only a negligible area of tumor necrosis was noticeable in its central part. The overall survival in the control group was 42 days, and in the study group 116 days [108].

### 5.7. Dermatological and Skin Indications

5-ALA-PDT is a widely used therapeutic method, not only in the treatment of cancerous lesions but also for numerous dermatological conditions, such as difficult-to-heal wounds [109], skin infections [110], condyloma acuminata [111,112], actinic keratosis [113,114], acne vulgaris [115], rosacea [116], cystic acne [117], hidradenitis suppurativa [118], and lichen sclerosus vulvae [119].

In the context of actinic keratosis, it is important to emphasize that daylight PDT (d-PDT) is currently a recognized alternative to conventional PDT. Meta-analyses have demonstrated equivalent therapeutic efficacy with a significantly better tolerability profile, including significantly less pain—approximately 79% of patients report no significant discomfort. For this reason, this method was included in the recommendations of the European Dermatology Forum (EDF, 2019 guidelines [120]) as the preferred treatment option for actinic keratosis.

Currently, skin cancer is one of the most common cancers worldwide, and the challenge of its treatment is constantly growing. It is estimated that approximately 5.4 million new cases are diagnosed annually in the United States. Skin cancers are divided into two main groups: melanomas derived from melanocytes and non-melanocytic epidermal tumors [121]. Surgery remains the standard treatment, but cryotherapy and pharmacological treatments, including 5-fluorouracil, are also important [114].

With regard to melanoma, it should be noted that classic PDT is limited or contraindicated due to the strong absorption of light by melanin, which reduces the efficiency of reactive oxygen species generation. At the same time, alternative approaches are being developed in preclinical models, such as SDT with 5-ALA, which in mouse models of skin melanoma showed significant inhibition of tumor growth of approximately 86.5% (B16-F10 mouse model), confirming its strong antitumor potential in preclinical conditions [122]. Wang et al. used 5-ALA-PDT on 76 patients with confirmed skin cancer or premalignant conditions. Most of the following conditions were cured or at least in partial remission: squamous cell carcinoma, basal cell carcinoma, Bowen’s disease, Paget’s disease, actinic keratosis, and erythroplasia of Queyrat. The best response was observed in actinic keratosis, Bowen’s disease, and superficial basal cell carcinoma, with poorer results observed in nodular basal cell carcinoma and squamous cell carcinoma [123]. Cordoba et al. studied the effect of 5-ALA-PDT on Mel25 cells derived from mouse skin melanoma, the mouse fibroblast cell line NIH3T3, the human melanoma cell line A375, and the mouse B16-F0. All cell lines showed a response to treatment with the following parameters: radiation intensity of 150 mW/cm^2^, energy doses of 45 and 90 J/cm^2^, and 5-ALA concentrations of 50 and 100 μg/mL. The B16-F0 line was the most susceptible to therapy; the mortality rate was similar at both 5-ALA concentrations and was approximately 92%. A better effect was not achieved after the use of 200 μg/mL 5-ALA and an energy dose of 180 J/cm^2^. The A375 and NIH3T3 cell lines showed a lower survival rate after the use of a higher 5-ALA concentration. The survival rate of the B16-F0, A375 and NIH3T3 cell lines was lower with the use of a higher radiation dose, regardless of the ALA concentration. Mel25 cells showed a mortality rate of 60–80% at 90 J/cm^2^ at both 5-ALA concentrations, which was higher than that at 45 J/cm^2^. In vivo studies showed that 5-ALA-PDT had no effect on the metastatic capacity of MT-ret [124].

### 5.8. Summary of Evidence and Limitations

Overall, the clinical relevance of 5-ALA-PDT varies substantially depending on tumor type, anatomical location, and the level of available evidence. The strongest clinical support exists for selected light-accessible lesions and fluorescence-guided applications, particularly in neuro-oncology, dermatology, urology, and selected premalignant mucosal lesions. In contrast, breast cancer, ovarian cancer, prostate cancer, and several gastrointestinal malignancies are still supported mainly by preclinical or early translational evidence [50,51,53,74,76,92].

Despite promising clinical and experimental results, several important limitations of 5-ALA-mediated PDT should be considered. The first major limitation is the restricted penetration depth of light in biological tissues. This makes PDT particularly effective in superficial or surgically accessible lesions, but limits its use in deep-seated, bulky, or diffusely infiltrating tumors. Therefore, tumor location and accessibility to adequate irradiation remain key determinants of therapeutic efficacy [3,53].

Another important limitation is tumor hypoxia. Since the photodynamic effect depends largely on oxygen availability and the generation of reactive oxygen species, poorly oxygenated tumor regions may respond less effectively to PDT. This is particularly relevant in aggressive solid tumors, where hypoxic areas are common and may contribute to treatment resistance [3,39,55].

The effectiveness of 5-ALA-PDT also depends on heterogeneous intracellular accumulation of protoporphyrin IX (PpIX). PpIX levels may vary between tumor types, between patients, and even between different areas of the same tumor. This variability is influenced by differences in heme biosynthesis, ferrochelatase activity, iron metabolism, ABC transporter expression, and 5-ALA uptake. As a result, fluorescence intensity and phototoxic response may not always correlate uniformly with tumor burden or treatment efficacy [21,42,43,50,53].

A further limitation is the heterogeneous level of available evidence. While some applications, such as fluorescence-guided glioma surgery, selected dermatological indications, and certain premalignant mucosal lesions, are supported by clinical data, many other indications remain based mainly on in vitro studies, animal models, or early clinical observations. Therefore, results obtained in breast cancer, ovarian cancer, prostate cancer, and several gastrointestinal tumors should be interpreted with caution until confirmed in larger clinical trials [10,50,51,77,79,88,89,96,101,105,106,107,108].

The lack of standardized treatment parameters is another important challenge. Studies differ in terms of 5-ALA concentration or dose, route of administration, incubation time, wavelength, fluence, irradiance, number of treatment sessions, and timing of outcome assessment. These methodological differences make direct comparison between studies difficult and limit the possibility of defining universal therapeutic protocols [10,47,48,50,53].

Finally, translation from in vitro studies to clinical practice remains limited. Cell culture models do not fully reproduce tumor architecture, vascularization, immune response, stromal interactions, oxygen gradients, or drug distribution in living tissues. Consequently, promising in vitro phototoxic effects may not always translate into comparable clinical efficacy. Further well-designed translational studies and randomized clinical trials are needed to determine the real therapeutic value of 5-ALA-PDT in specific cancer types [10,50,55,56,77].

An overview of the preclinical, clinical, and translational evidence on 5-ALA-mediated PDT is presented in Table 3.

Importantly, substantial intertumoral differences exist in both the clinical applicability and therapeutic efficacy of 5-ALA-based approaches. In glioblastoma, 5-ALA has established clinical value primarily as a fluorescence-guided surgical adjunct, where selective PpIX accumulation enables improved tumor visualization and more complete resection, while evidence for direct therapeutic PDT remains more limited but promising. Dermatological indications, including actinic keratosis, superficial basal cell carcinoma, and selected premalignant skin lesions, generally demonstrate favorable outcomes due to direct light accessibility, relatively predictable PpIX accumulation, and established treatment protocols. Similarly, urological applications such as bladder cancer remain clinically feasible because intraluminal light delivery can be performed relatively easily, partially overcoming one of the major physical limitations of PDT. In contrast, deep-seated solid tumors such as pancreatic, prostate, ovarian, and many gastrointestinal malignancies present substantially greater therapeutic challenges due to restricted light penetration, tumor hypoxia, heterogeneous porphyrin metabolism, and variable biological responses. Breast cancer represents an intermediate case, where promising in vitro findings—particularly in triple-negative subtypes—suggest therapeutic potential, but robust clinical validation remains lacking. These differences collectively indicate that the clinical utility of 5-ALA-based PDT is highly tumor-dependent and should not be generalized uniformly across all oncological indications [125,126,127,128].

## 6. 5-ALA Combination Therapies in Oncology: Mechanisms, Resistance, and New Directions for Development

Currently, clinical evidence supporting the combination of 5-ALA-PDT with other therapeutic approaches remains limited and indication-dependent. The most advanced clinical data are available for glioblastoma, where intraoperative 5-ALA-PDT has been investigated as an adjunct to maximal safe surgical resection and standard postoperative treatment. In this context, the INDYGO trial demonstrated the feasibility and safety of standardized intraoperative 5-ALA-PDT after resection, suggesting that PDT may help target residual tumor cells within the resection cavity. However, most other combination strategies, including chemotherapy, targeted therapy, immunotherapy, nanocarrier-based delivery systems, radiodynamic therapy, and sonodynamic therapy, are currently supported mainly by preclinical, mechanistic, or early translational evidence rather than robust clinical trial data. Therefore, these approaches should be regarded as promising but not yet clinically validated, and further prospective controlled studies are required to determine their therapeutic value across specific tumor types [79,125,129].

### 6.1. PDT—5-ALA, PpIX, and ROS (Modern Approach)

The most established therapeutic approach using 5-ALA is its use in PDT, which forms the basis of the clinical and experimental use of this strategy in oncology. 5-ALA is a precursor in the biosynthesis of PpIX, which selectively accumulates in cancer cells due to disruption of the heme pathway. When excited by light of a specific wavelength, PpIX generates ROS, leading to oxidative stress and damage to cellular structures, including mitochondria and DNA, resulting in cancer cell death through apoptosis and necrosis [50,130].

### 6.2. SDT—Ultrasound as an Extension of PDT (Very Current)

A significant and rapidly developing extension of classic PDT is SDT, which uses ultrasound, including high-intensity focused ultrasound (FUS), instead of light. The mechanism of SDT involves cavitation and mechanical effects, which lead to the activation of sonosensitizers and the generation of ROS. Due to its high tissue penetration and ability to pass through bone structures, SDT is a particularly promising treatment strategy for brain tumors, including glioblastoma multiforme, while classic PDT is limited by insufficient light penetration [131].

SDT, like PDT, is based on the induction of oxidative stress, including ROS generation through sonoluminescence, cavitation, and thermal effects. The resulting oxidative stress leads to DNA damage, mitochondrial dysfunction, and the activation of cell death pathways, primarily apoptosis. Additionally, the cytotoxic effect of SDT may be enhanced by local hyperthermia and mechanical tissue damage induced by microbubble oscillation, which further increases the production of hydroxyl radicals and ROS [132]. Studies have also shown that SDT can exhibit immunomodulatory and antiangiogenic effects, including by inhibiting endothelial cell proliferation and migration [133,134].

**Figure 7:**
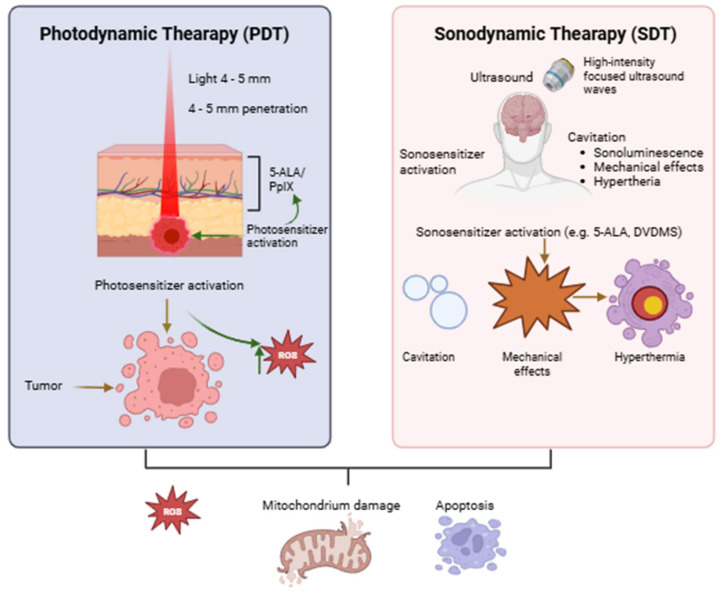
Comparison of PDT and SDT. PDT is based on the activation of a photosensitizer (e.g., 5-aminolevulinate, 5-ALA, leading to the formation of protoporphyrin IX, PpIX) with light, which results in the generation of ROS and the induction of cancer cell death. Due to the limited light penetration, this method is mainly used in the treatment of superficial lesions. Sonodynamic therapy (SDT) utilizes ultrasound, including high-intensity focused ultrasound (FUS), which enables deep tissue penetration, including in brain structures. The mechanism of action of SDT involves cavitation, sonoluminescence, and mechanical and thermal effects, leading to the activation of sonosensitizers (e.g., 5-ALA, DVDMS) and increased ROS production. Both methods lead to mitochondrial damage, oxidative stress and activation of apoptotic pathways, but SDT has an advantage in the treatment of deep-seated and diffuse tumors due to its greater ability to penetrate tissues.

Preclinical experiments have used a wide range of ultrasound parameters, including intensities of 0.2–25 W/cm^2^, frequencies of 0.5–3 MHz, and exposure times from 10 ms to 20 min. Among the sonosensitizers tested are 5-ALA, fluorescein (FL), sodium sinoporphyrin (DVDMS), hematoporphyrin monomethyl ether (HMME), and Photofrin [135], with 5-ALA having one of the best-documented biocompatibility and clinical safety profiles [136]. The advantage of SDT over PDT is its ability to effectively target both deep-seated and diffuse lesions, which results from improved tissue penetration of ultrasound (Figure 7). Both in vitro studies and in vivo models confirm the efficacy of SDT through the induction of apoptosis and ROS generation, supporting its potential as a translational strategy in cancer treatment [135,137,138,139,140].

### 6.3. Fluorescence-Guided Surgery (Clinical Standard for GBM)

In clinical practice, 5-ALA finds key applications in fluorescence-guided surgery (FGS), where oral administration selectively accumulates PpIX in tumor cells. Fluorescence emitted by PpIX under the influence of blue light enables real-time intraoperative tumor visualization, allowing the surgeon to more accurately distinguish the tumor from healthy tissue. This mechanism is based on impaired heme metabolism in tumor cells, leading to increased PpIX accumulation, and forms the basis for 5-ALA’s use as a theranostic tool in neuro-oncology [141].

The use of 5-ALA in glioma surgery translates directly into improved extent of resection (EOR), which is one of the most important prognostic factors in these tumors. Systematic studies and meta-analyses indicate that fluorescence-guided surgery enables higher rates of gross total resection (GTR) and is associated with improved overall survival and progression-free survival compared to white light surgery. At the same time, this method preserves neurological function by more precisely delineating tumor boundaries and limiting damage to healthy tissue [126,142].

This technique is particularly important in glial tumors, which are characterized by infiltrative growth and a lack of distinct anatomical boundaries. In such cases, conventional surgery often fails to completely remove the lesion, but the use of 5-ALA significantly improves the visualization of infiltrating tumor margins. Recent systematic reviews also confirm the safety of this method and its growing use in other types of brain tumors, although the effectiveness of fluorescence may vary depending on the degree of malignancy and histological type. Consequently, fluorescence-guided surgery using 5-ALA has become a standard supporting maximum safe resection in modern oncological neurosurgery [143,144].

### 6.4. Biological Limitations of 5-ALA-PDT: Hypoxia, Heterogeneous PpIX Accumulation, Resistance Mechanisms, and Tumor-Specific Variability

One of the major biological limitations of 5-ALA-mediated photodynamic therapy is tumor hypoxia. Since the cytotoxic effect of PDT depends largely on the generation of reactive oxygen species following activation of PpIX, sufficient oxygen availability is essential for therapeutic efficacy. However, many aggressive solid tumors, including glioblastoma, pancreatic cancer, and advanced head and neck malignancies, contain hypoxic regions resulting from abnormal vascularization, rapid cellular proliferation, and impaired oxygen diffusion. These hypoxic microenvironments significantly reduce ROS generation and therefore weaken the photodynamic effect. In addition, hypoxia may promote adaptive survival signaling, metabolic reprogramming, and treatment resistance, further limiting the clinical effectiveness of PDT [3,55,131,145].

A further clinically relevant limitation is the restricted penetration of light into biological tissues. The efficacy of 5-ALA-PDT depends not only on intracellular PpIX accumulation, but also on whether sufficient light energy can reach the entire tumor volume. For this reason, superficial, endoscopically accessible, or surgically exposed lesions are generally more suitable for PDT than deep-seated or bulky tumors. In larger or deeply infiltrating tumors, the peripheral tumor area may be adequately irradiated, whereas deeper regions may receive an insufficient light dose, leading to incomplete PpIX activation and heterogeneous tumor cell destruction. This physical limitation partly explains why 5-ALA-PDT shows stronger clinical applicability in dermatological, mucosal, bladder, and intraoperatively exposed brain lesions, while its use in deeply located solid tumors remains more challenging [3,10,53,131].

Bordoloi et al., 2024, indicate that limited light penetration and the heterogeneous response resulting from varying penetration depths are significant limitations of 5-ALA-PDT [146]. Hua et al., 2024, describe shallow light penetration as one of the main challenges of PDT in the treatment of deep tumors [147]. Bader et al., 2025, directly address the assessment of the depth of action of 5-ALA-PDT in the GBM model [148].

Another major limitation is the heterogeneous intracellular accumulation of PpIX, which represents a key determinant of both photodynamic efficacy and fluorescence-guided diagnostics. PpIX accumulation varies not only between different tumor entities, but also between individual patients and even between distinct regions of the same tumor. This heterogeneity is influenced by multiple biological factors, including differences in heme biosynthesis, ferrochelatase activity, iron metabolism, peptide transporter expression, ATP-binding cassette transporter activity, and broader metabolic adaptations. Consequently, fluorescence intensity does not always correlate uniformly with tumor burden, histological aggressiveness, or therapeutic response, complicating both diagnostic interpretation and treatment predictability [21,42,43,50,53,149,150].

The expression and activity of membrane transporters constitute another important biological limitation. Among these, ABCG2 remains one of the most extensively studied mechanisms, reducing intracellular PpIX accumulation through active efflux. Increased ABCG2 expression decreases PpIX retention, reduces fluorescence intensity, and weakens PDT-mediated cytotoxicity. However, transporter-mediated resistance appears to be more complex than the activity of a single protein. Other ATP-binding cassette transporters, alternative PpIX export mechanisms, and tumor-specific transport dynamics may also contribute to treatment variability. Furthermore, studies suggest that mechanisms such as dynamin-dependent exocytosis may participate in regulating intracellular porphyrin levels independently of classical ABC transporter pathways [44,151,152,153,154,155].

Resistance to 5-ALA-PDT may also develop through alterations in the heme biosynthetic pathway itself. Reduced activity of ferrochelatase, altered porphobilinogen deaminase expression, dysregulated coproporphyrinogen oxidase activity, and disturbances in iron homeostasis may significantly affect PpIX generation and retention. More recent evidence suggests that repeated PDT exposure may induce acquired resistance through downregulation of enzymes involved in porphyrin synthesis, reducing intracellular photosensitizer availability despite adequate 5-ALA administration. This suggests that resistance mechanisms are not limited to transporter overexpression, but may involve broader metabolic adaptation [21,33,34,46,50,149].

The most clinically relevant mechanisms of tumor resistance or escape from 5-ALA-PDT include insufficient intracellular PpIX accumulation, enhanced porphyrin efflux through ABC transporters such as ABCG2 and ABCB1, adaptive remodeling of the heme biosynthetic pathway, tumor hypoxia, increased antioxidant and stress-response capacity, autophagy-mediated survival, and cancer stem cell–associated resistance. Importantly, resistance to 5-ALA-PDT should not be interpreted solely as a consequence of increased PpIX efflux. Recent evidence suggests that acquired resistance may also develop through reduced activity or expression of enzymes involved in porphyrin synthesis, including PBGD, even when ABCG2 inhibition is applied. In addition, cancer stem cell populations may survive 5-ALA-PDT more effectively due to lower PpIX accumulation, reduced ROS-mediated damage, increased transporter activity, and enhanced survival programs. These mechanisms collectively support the need for combination strategies targeting not only photosensitizer accumulation, but also tumor metabolism, oxidative stress adaptation, stem-like cell populations, and the tumor microenvironment [45,46,153,156].

Cancer stem cells represent an additional clinically relevant challenge. These subpopulations frequently demonstrate intrinsic resistance to 5-ALA-PDT due to lower intracellular PpIX accumulation, enhanced efflux transporter activity, and altered metabolic profiles. Because cancer stem cells are believed to contribute to tumor recurrence, progression, and therapeutic resistance, their reduced susceptibility to PDT may significantly limit long-term treatment efficacy. In contrast, dormant tumor cell populations may display different porphyrin accumulation behavior, highlighting the complexity of tumor heterogeneity [45,157].

Importantly, these biological limitations are highly tumor-dependent. While 5-ALA has demonstrated strong clinical utility in fluorescence-guided glioma surgery, selected dermatological applications, and some premalignant mucosal lesions, other tumor types such as breast, ovarian, prostate, and gastrointestinal malignancies show far more variable and less predictable responses. These differences likely result from tumor-specific metabolic programs, oxygenation status, transporter expression profiles, stromal interactions, and microenvironmental factors. Therefore, extrapolation of results between tumor types should be approached cautiously [10,50,51,77,88,89,96,101,105,106,107,108,156,158].

Taken together, these limitations demonstrate that the efficacy of 5-ALA-based PDT and PDD depends not only on light exposure and photosensitizer administration, but also on a complex interplay of tumor biology, metabolism, oxygen availability, and resistance mechanisms. A better understanding of these processes is essential for improving patient selection, optimizing treatment protocols, and developing combination strategies capable of overcoming current biological barriers [3,50,53,55,56,131,150,153].

From a clinical perspective, the main barriers limiting broader implementation of 5-ALA-PDT include insufficient light penetration, tumor hypoxia, heterogeneous PpIX accumulation, variable tumor-specific biology, non-standardized treatment protocols, and emerging resistance mechanisms. Future optimization will likely require interstitial or image-guided light delivery, improved 5-ALA formulations and prodrugs, nanocarrier-based delivery systems, oxygen-modulating strategies, transporter or heme-pathway modulation, and rational combination therapies. A comparative overview of the current clinical applications, major limitations, and future development directions of 5-ALA/PpIX-based approaches across different oncological indications is presented in Table 4.

### 6.5. Targeted Therapies and Multipathway Synergy

Another important area is the combination of 5-ALA with signaling pathway inhibitors, particularly MEK. Therapeutic synergy results from simultaneously affecting various levels of regulation of cancer cell proliferation and survival, which allows for limiting the mechanisms of compensatory activation of survival pathways. In particular, blocking the MAPK axis (RAF-MEK-ERK) may increase cell susceptibility to oxidative stress induced by 5-ALA-PDT and enhance the cytotoxic effect by disrupting proliferative homeostasis [163].

Additionally, combinations combining BRAF or mTOR inhibitors are important, as they counteract the activation of alternative cancer cell survival pathways and limit MAPK reactivation. Simultaneous inhibition of the MAPK and PI3K/AKT/mTOR axis leads to a more comprehensive inhibition of cancer cell proliferation and survival, which may enhance the effects of photodynamic therapy and limit the development of treatment resistance [164].

In RAS-mutated cancers, a particularly significant synergistic effect is observed between MEK therapy and PARP inhibitors, leading to increased apoptosis through BIM activation and the accumulation of DNA damage, resulting in impaired repair mechanisms and increased cell death. This approach aligns with the concept of multipathway therapy, in which simultaneous inhibition of proliferation, DNA repair, and survival pathways allows for more effective tumor growth reduction [165,166,167].

### 6.6. Immunogenic Cell Death and the Vaccine Approach to PDT and Extracorporeal Therapy

A significant area of research is the use of 5-ALA-PDT to induce immunogenic cell death (ICD), which enables the transformation of immunologically “cold” tumors into “hot” ones, making them more susceptible to an immune response. ICD is characterized by the release of danger signaling molecules (DAMPs) such as calreticulin, ATP, and HMGB1, which lead to the activation of dendritic cells and the initiation of a T-cell response. In the case of 5-ALA-PDT, this process is a consequence of severe oxidative stress induced by reactive oxygen species (ROS), which leads to both tumor cell death (apoptosis and necrosis) and increased immunogenicity [168,169].

The immunogenic effect of 5-ALA-PDT can be significantly enhanced by modulating cell survival pathways, particularly by inhibition of the AKT axis. AKT inhibition promotes the exposure of calreticulin on the cell surface and increased release of ATP and HMGB1, resulting in more effective activation of antigen-presenting cells and a stronger cytotoxic T lymphocyte response. This mechanism translates not only into control of primary tumor growth but also inhibition of micrometastases and systemic effects. These data support the concept that 5-ALA-PDT can act as an “in situ vaccine,” especially when combined with checkpoint inhibitors such as anti-PD-1 and anti-PD-L1, which further reverse the immunosuppression of the tumor microenvironment and enhance a sustained antitumor response [165,166,167,170,171].

An extension of this concept is extracorporeal PDT, in which blood cells or tumor cells are exposed to 5-ALA-PDT outside the body and then reintroduced into circulation. In this model, controlled induction of ICD leads to the release of tumor antigens and DAMPs, resulting in dendritic cell activation and a potent T-cell-dependent adaptive response. Preclinical models have shown that this approach can act as a “vaccine-like” therapy, inducing a systemic antitumor response and enabling control of both the primary tumor and distant lesions [172].

Current research is focused on optimizing this technology by developing systems that combine blood flow with PDT, particularly in the context of hematological malignancies. Key directions include increasing tissue oxygenation (oxygen-boosted PDT), using nanoparticles to enhance PpIX accumulation, and personalizing therapy based on the tumor’s metabolic and immunological profile. These strategies aim to increase the efficacy of ICD induction and enhance the immune response, potentially leading to the emergence of a new class of systemic immunophotodynamic therapies [168,172].

## 7. Conclusions

An analysis of the available literature indicates that 5-ALA is a key element of contemporary diagnostic and therapeutic strategies in cancer PDT and PDD. Its particular value stems from its prodrug activity; it is converted in cancer cells to PpIX, which exhibits both fluorescent and phototoxic properties.

Accumulated data confirm that the effectiveness of 5-ALA-PDT is strongly dependent on tumor characteristics, the activity of the heme biosynthetic pathway, and the tumor microenvironment. The best clinical outcomes are observed in superficial and light-accessible tumors, such as skin lesions, bladder cancer, and selected head and neck tumors. High therapeutic response rates are observed in many indications, particularly in the early stages of the disease.

At the same time, preclinical study results highlight significant limitations of the method, including heterogeneity in PpIX accumulation, the impact of tumor hypoxia, and the differential expression of transporters and enzymes of the hemin pathway (e.g., FECH, PBGD, CPOX, and PEPT1/2). Increasing evidence suggests that the tumor microenvironment plays a direct and clinically relevant role in determining the efficacy of 5-ALA-PDT. Hypoxic tumor regions may substantially reduce reactive oxygen species generation, thereby weakening the photodynamic effect despite adequate photosensitizer administration. Abnormal tumor vascularization may further impair oxygen delivery and limit homogeneous distribution of 5-ALA within the tumor. In addition, stromal interactions, including those involving cancer-associated fibroblasts, may alter porphyrin metabolism and contribute to intertumoral variability in PpIX accumulation. Metabolic adaptation, antioxidant defense mechanisms, and survival signaling within the tumor microenvironment may further promote resistance to oxidative stress-induced cell death. In this context, rational combination strategies may improve clinical efficacy by addressing these biological barriers—for example, through oxygen-modulating approaches, transporter inhibition, heme-pathway modulation, targeted therapies, immunotherapy-based combinations, or advanced delivery systems designed to improve tumor selectivity and photosensitizer retention.

The development of resistance mechanisms, which are not limited to ABC transporters (e.g., ABCG2) but also encompass changes in the activity of enzymes of the heme biosynthesis pathway, including PBGD reduction, remains a significant clinical problem. This indicates the need for a more comprehensive approach to monitoring and modulating porphyrin metabolism during therapy.

Concurrently, the rapid development of nanotechnology, chemical modifications of 5-ALA, and drug delivery systems is significantly expanding the clinical potential of this strategy. The use of nanometric carriers, prodrugs, and combination therapies (e.g., PTT, SDT, RDT) allows for increased selectivity, improved bioavailability, and overcoming limitations related to tumor hypoxia.

In summary, 5-ALA-PDT remains a method with significant translational potential, particularly for the treatment of superficial tumors and in fluorescent surgical techniques. Its further development will likely depend on the integration of molecular approaches that consider porphyrin metabolism and the tumor microenvironment, as well as the implementation of advanced delivery systems and combination therapies.

## Figures and Tables

**Table 1:** Causes of PpIX accumulation in cells.

1.	Increase in 5-ALA level
2.	Hyperactivity of 5-ALA synthase
3.	Dysfunction of the ferrochelatase

**Table 2:** Factors that may influence the in vitro sensitivity of cancer cells [3,47,48].

FACTORS
timing of irradiation
duration between irradiation and viability assays
wavelength of irradiation
fluence
components of 5-ALA
concentration of 5-ALA
initial cell density
washing conditions
incubation time
light irradiance

**Table 3:** Overview of preclinical, clinical, and translational evidence on 5-ALA-mediated PDT across major oncological and dermatological disease entities, including study models, levels of evidence, and key therapeutic outcomes.

Clinical Area/Disease	Evidence Status	Model/Population	Key Findings	References
Glioblastoma multiforme (GBM)	Clinical + preclinical	GBM patients, U87, GIC7, PG88 models	Improved survival (OS ~23.1 months), high selective PpIX accumulation, light-dose-dependent apoptosis induction	[77,78,79]
Medulloblastoma	Preclinical	MB lines Med8A, UW228-2, ONS76	Increased apoptosis dependent on 5-ALA concentration and incubation time	[80]
Meningioma	Preclinical	Primary cultures	Cell viability decreased to ~13.8% at 100 μg/mL	[81]
Head and neck cancer	Clinical + Phase I	Patients, precancerous lesions	92% response, CR up to 69% in early lesions	[86,87,88]
Breast cancer (TNBC, MCF-7)	Primarily in vitro	MCF-7, MDA-MB-231	Higher fluorescence and sensitivity in TNBC; no clinical data	[86,87,88,89]
Gynecological cancers (HSIL, CIN, VaIN)	clinical	HPV+ patients	HSIL regression up to 90.9%, HPV elimination ~86%	[92,94,95]
Ovarian cancer (cell lines)	Preclinical	OVMANA, RMG1, ES2, etc.	Dose-dependent cytotoxicity, IC50 56–882 μM	[96]
Bladder cancer	Clinical	10 patients	40% complete remission	[79]
Prostate cancer (radiodynamic therapy)	Preclinical	PC-3 mouse model	Tumor growth slowed by ~39%	[101]
Pancreatic cancer	Preclinical	Hamster model	Survival extension: 42 vs. 116 days	[108]
Dermatological conditions (non-cancerous)	Clinical	Dermatological patients	Wide range of applications (acne, AK, infections, etc.)	[109,110,111,112,113,114,115,116,117,118,119]
Skin cancer (general)	Clinical	Large series of patients	Good response in AK, superficial BCC	[117,124]
Melanoma (PDT limitations)	Preclinical	B16-F10 model	~86.5% growth inhibition in SDT (ALA)	[122]
Barrett’s esophagus (HGD)	Historical clinical	64 patients	55% vs. 22% remission (ALA vs. Photofrin), currently RFA standard	[103]
Colon/liver cancer	Preclinical	HT-29, HuH7, HepG2, etc.	Induction of apoptosis and inhibition of proliferation	[105,106,107]

**Table 4:** Comparative summary of clinical applications, major limitations, future perspectives, and representative references for 5-ALA/PpIX-based approaches in oncology.

Clinical Area	Current Clinical Relevance	Main Limitations	Future Perspectives	References
Glioblastoma/high-grade glioma	Established use in fluorescence-guided surgery; emerging intraoperative PDT	Tumor infiltration, heterogeneous fluorescence, limited PDT penetration, recurrence	Interstitial/intraoperative PDT, SDT, RDT, improved dosimetry, combination with standard therapy	[12,77,79,125]
Dermatological lesions	Established use in actinic keratosis, superficial BCC and selected premalignant lesions	Pain, recurrence, limited efficacy in thicker/nodular tumors	Daylight PDT, improved formulations, combination topical therapies	[113,114,128,159]
Bladder cancer/urothelial lesions	Clinically feasible due to intraluminal access	Recurrence, heterogeneous PpIX accumulation, variable response	Improved intravesical delivery, repeated protocols, optimized light delivery	[17,99,127]
Head and neck premalignant lesions	Promising clinical evidence in selected superficial mucosal lesions	Anatomical complexity, pain, recurrence, oxygenation variability	Image-guided PDT, oxygen monitoring, combination with local therapies	[84,85,146,160]
Gynecological premalignant lesions	Promising clinical outcomes in CIN/VaIN/HSIL	Need for longer follow-up, recurrence, HPV persistence	Standardized protocols, HPV-directed monitoring, fertility-sparing approaches	[92,94,95]
Breast cancer	Mainly preclinical; promising in TNBC models	Lack of robust clinical data, deep tissue location, heterogeneity	Combination with chemotherapy/targeted therapy, nanocarriers, intraoperative use	[88,89,161]
Ovarian/prostate/pancreatic/GI cancers	Mostly preclinical or early translational	Deep location, hypoxia, limited light access, variable PpIX metabolism	SDT/RDT, interstitial PDT, nanocarriers, oxygen-generating systems	[96,101,106,107]
Combination therapies	Conceptually promising; limited clinical validation	Mostly preclinical evidence, safety and standardization issues	Immunotherapy combinations, transporter inhibition, heme-pathway modulation, advanced delivery systems	[45,46,162]

## Data Availability

No new data were created or analyzed in this study.

## Abbreviations

5-ALA	pentaaminolevulinic acid
ABCB6	ATP-binding cassette transporter B6
ALAD	ALA dehydratase
ALAS	aminolevulinic acid synthase
CIN	cervical intraepithelial neoplasia
CPG I, CPG III	coproporphyrinogen I, coproporphyrinogen III
CPOX	coproporphyrinogen III oxidase
EDT	electrodynamic therapy
FECH	ferrochelatase
GBM	glioblastoma multiforme
HGG	high-grade glioma
HMB	hydroxymethylbilane
HPV	Human Papillomavirus
HSIL	High-grade Squamous Intraepithelial Lesion
IC50	half maximal inhibitory concentration
LGG	low-grade glioma
MDT	microwave dynamic therapy
PBG	porphobilinogen
PBGD	porphobilinogen deaminase
PDD	photodynamic diagnosis
PDT	photodynamic therapy
PEPT1, PEPT2	peptide transporter 1, peptide transporter 2
Ppgen	protoporphyrinogen
PPOX	protoporphyrinogen oxidase
PS	photosensitizer
RDT	radiodynamic therapy
ROS	reactive oxygen species
SDT	sonodynamic therapy
UPG III	uroporphyrinogen III
UROD	uroporphyrinogen decarboxylase
UROS	uroporphyrinogen III synthase
VaIN	vaginal intraepithelial neoplasia
VIN	vulvar intraepithelial neoplasia

## References

[1] Siegel R.L., Giaquinto A.N., Jemal A. (2024). Cancer statistics, 2024. CA Cancer J. Clin..

[2] Bray F., Laversanne M., Sung H., Ferlay J., Siegel R.L., Soerjomataram I., Jemal A. (2024). Global cancer statistics 2022: GLOBOCAN estimates of incidence and mortality worldwide for 36 cancers in 185 countries. CA Cancer J. Clin..

[3] Pignatelli P., Umme S., D’Antonio D.L., Piattelli A., Curia M.C. (2023). Reactive Oxygen Species Produced by 5-Aminolevulinic Acid Photodynamic Therapy in the Treatment of Cancer. Int. J. Mol. Sci..

[4] dos Santos A.F., de Almeida D.R.Q., Terra L.F., Baptista M.S., Labriola L. (2019). Photodynamic therapy in cancer treatment—An update review. J. Cancer Metastasis Treat..

[5] Correia J.H., Rodrigues J.A., Pimenta S., Dong T., Yang Z. (2021). Photodynamic Therapy Review: Principles, Photosensitizers, Applications, and Future Directions. Pharmaceutics.

[6] Goldman M., Atkin D. (2003). ALA/PDT in the treatment of actinic keratosis: Spot versus confluent therapy. J. Cosmet. Laser Ther..

[7] Zhou Z., Song J., Nie L., Chen X. (2016). Reactive oxygen species generating systems meeting challenges of photodynamic cancer therapy. Chem. Soc. Rev..

[8] Lee C.N., Hsu R., Chen H., Wong T.W. (2020). Daylight Photodynamic Therapy: An Update. Molecules.

[9] Wan W., Liu H., Zou J., Xie T., Zhang G., Ying W., Zou X. (2025). The optimization and application of photodynamic diagnosis and autofluorescence imaging in tumor diagnosis and guided surgery: Current status and future prospects. Front. Oncol..

[10] Shinoda Y., Kato D., Ando R., Endo H., Takahashi T., Tsuneoka Y., Fujiwara Y. (2021). Systematic Review and Meta-Analysis of In Vitro Anti-Human Cancer Experiments Investigating the Use of 5-Aminolevulinic Acid (5-ALA) for Photodynamic Therapy. Pharmaceuticals.

[11] Weller M., van den Bent M., Preusser M., Le Rhun E., Tonn J.C., Minniti G., Bendszus M., Balana C., Chinot O., Dirven L. (2021). EANO guidelines on the diagnosis and treatment of diffuse gliomas of adulthood. Nat. Rev. Clin. Oncol..

[12] Stummer W., Pichlmeier U., Meinel T., Wiestler O.D., Zanella F., Reulen H.J. (2006). ALA-Glioma Study Group. Fluorescence-guided surgery with 5-aminolevulinic acid for resection of malignant glioma: A randomised controlled multicentre phase III trial. Lancet Oncol..

[13] Yonemura Y., Endo Y., Canbay E., Liu Y., Ishibashi H., Mizumoto A., Hirano M., Imazato Y., Takao N., Ichinose M. (2017). Photodynamic Detection of Peritoneal Metastases Using 5-Aminolevulinic Acid (ALA). Cancers.

[14] Dolmans D.E., Fukumura D., Jain R.K. (2003). Photodynamic therapy for cancer. Nat. Rev. Cancer.

[15] Thunshelle C., Yin R., Chen Q., Hamblin M.R. (2016). Current Advances in 5-Aminolevulinic Acid Mediated Photodynamic Therapy. Curr. Dermatol. Rep..

[16] Zeitouni N.C., Oseroff A.R., Shieh S. (2003). Photodynamic therapy for nonmelanoma skin cancers. Current review and update. Mol. Immunol..

[17] Inoue K., Fukuhara H., Yamamoto S., Karashima T., Kurabayashi A., Furihata M., Hanazaki K., Lai H.W., Ogura S.I. (2022). Current status of photodynamic technology for urothelial cancer. Cancer Sci..

[18] Beika M., Harada Y., Minamikawa T., Yamaoka Y., Koizumi N., Murayama Y., Konishi H., Shiozaki A., Fujiwara H., Otsuji E. (2021). Accumulation of Uroporphyrin I in Necrotic Tissues of Squamous Cell Carcinoma after Administration of 5-Aminolevulinic Acid. Int. J. Mol. Sci..

[19] Holgersen E.M., Gandhi S., Zhou Y., Kim J., Vaz B., Bogojeski J., Bugno M., Shalev Z., Cheung-Ong K., Gonçalves J. (2021). Transcriptome-Wide Off-Target Effects of Steric-Blocking Oligonucleotides. Nucleic Acid. Ther..

[20] Liu Z., Mela A., Argenziano M.G., Banu M.A., Furnari J., Kotidis C., Sperring C.P., Humala N., Mahajan A., Bruce J.N. (2023). Single-cell analysis of 5-aminolevulinic acid intraoperative labeling specificity for glioblastoma. J. Neurosurg..

[21] Traylor J.I., Pernik M.N., Sternisha A.C., McBrayer S.K., Abdullah K.G. (2021). Molecular and Metabolic Mechanisms Underlying Selective 5-Aminolevulinic Acid-Induced Fluorescence in Gliomas. Cancers.

[22] Allison R.R. (2014). Photodynamic therapy: Oncologic horizons. Future Oncol..

[23] Braathen L.R., Szeimies R.M., Basset-Seguin N., Bissonnette R., Foley P., Pariser D., Roelandts R., Wennberg A.M., Morton C.A. (2007). Guidelines on the use of photodynamic therapy for nonmelanoma skin cancer: An international consensus. J. Am. Acad. Dermatol..

[24] Blume J.E., Oseroff A.R. (2007). Aminolevulinic acid photodynamic therapy for skin cancers. Dermatol. Clin..

[25] Kaneko S., Kaneko S. (2016). Fluorescence-Guided Resection of Malignant Glioma with 5-ALA. Int. J. BioMed Imaging.

[26] Chen X., Wang C., Teng L., Liu Y., Chen X., Yang G., Wang L., Liu H., Liu Z., Zhang D. (2014). Calcitriol enhances 5-aminolevulinic acid-induced fluorescence and the effect of photodynamic therapy in human glioma. Acta Oncol..

[27] Krieg R.C., Messmann H., Rauch J., Seeger S., Knuechel R. (2002). Metabolic characterization of tumor cell-specific protoporphyrin IX accumulation after exposure to 5-aminolevulinic acid in human colonic cells. Photochem. Photobiol..

[28] Krieg R.C., Fickweiler S., Wolfbeis O.S., Knuechel R. (2000). Cell-type specific protoporphyrin IX metabolism in human bladder cancer in vitro. Photochem. Photobiol..

[29] Fukuhara H., Inoue K., Kurabayashi A., Furihata M., Fujita H., Utsumi K., Sasaki J., Shuin T. (2013). The inhibition of ferrochelatase enhances 5-aminolevulinic acid-based photodynamic action for prostate cancer. Photodiagnosis Photodyn. Ther..

[30] Navone N.M., Polo C.F., Frisardi A.L., Andrade N.E., Battle A.M. (1990). Heme biosynthesis in human breast cancer--mimetic "in vitro" studies and some heme enzymic activity levels. Int. J. Biochem..

[31] Hinnen P., de Rooij F.W., van Velthuysen M.L., Edixhoven A., van Hillegersberg R., Tilanus H.W., Wilson J.H., Siersema P.D. (1998). Biochemical basis of 5-aminolaevulinic acid-induced protoporphyrin IX accumulation: A study in patients with (pre)malignant lesions of the oesophagus. Br. J. Cancer.

[32] Schauder A., Feuerstein T., Malik Z. (2011). The centrality of PBGD expression levels on ALA-PDT efficacy. Photochem. Photobiol. Sci..

[33] Pustogarov N., Panteleev D., Goryaynov S.A., Ryabova A.V., Rybalkina E.Y., Revishchin A., Potapov A.A., Pavlova G. (2017). Hiding in the Shadows: CPOX Expression and 5-ALA Induced Fluorescence in Human Glioma Cells. Mol. Neurobiol..

[34] Sinha A.K., Anand S., Ortel B.J., Chang Y., Mai Z., Hasan T., Maytin E.V. (2006). Methotrexate used in combination with aminolaevulinic acid for photodynamic killing of prostate cancer cells. Br. J. Cancer.

[35] Adapa S.R., Hunter G.A., Amin N.E., Marinescu C., Borsky A., Sagatys E.M., Sebti S.M., Reuther G.W., Ferreira G.C., Jiang R.H. (2024). Porphyrin overdrive rewires cancer cell metabolism. Life Sci. Alliance.

[36] Moesta K.T., Ebert B., Handke T., Nolte D., Nowak C., Haensch W.E., Pandey R.K., Dougherty T.J., Rinneberg H., Schlag P.M. (2001). Protoporphyrin IX occurs naturally in colorectal cancers and their metastases. Cancer Res..

[37] Bellini M.H., Coutinho E.L., Courrol L.C., Rodrigues de Oliveira Silva F., Vieira Júnior N.D., Schor N. (2008). Correlation between autofluorescence intensity and tumor area in mice bearing renal cell carcinoma. J. Fluoresc..

[38] Algorri J.F., Ochoa M., Roldán-Varona P., Rodríguez-Cobo L., López-Higuera J.M. (2021). Photodynamic Therapy: A Compendium of Latest Reviews. Cancers.

[39] Gong Z., Dai Z. (2021). Design and Challenges of Sonodynamic Therapy System for Cancer Theranostics: From Equipment to Sensitizers. Adv. Sci. (Weinh).

[40] Bunevicius A., Pikis S., Padilla F., Prada F., Sheehan J. (2022). Sonodynamic therapy for gliomas. J. Neurooncol..

[41] Cheff D.M., Huang C., Scholzen K.C., Gencheva R., Ronzetti M.H., Cheng Q., Hall M.D., Arnér E.S.J. (2023). The ferroptosis inducing compounds RSL3 and ML162 are not direct inhibitors of GPX4 but of TXNRD1. Redox Biol..

[42] McNicholas K., MacGregor M.N., Gleadle J.M. (2019). In order for the light to shine so brightly, the darkness must be present-why do cancers fluoresce with 5-aminolaevulinic acid?. Br. J. Cancer.

[43] Harada Y., Murayama Y., Takamatsu T., Otsuji E., Tanaka H. (2022). 5-Aminolevulinic Acid-Induced Protoporphyrin IX Fluorescence Imaging for Tumor Detection: Recent Advances and Challenges. Int. J. Mol. Sci..

[44] Kitajima Y., Ishii T., Kohda T., Ishizuka M., Yamazaki K., Nishimura Y., Tanaka T., Dan S., Nakajima M. (2019). Mechanistic study of PpIX accumulation using the JFCR39 cell panel revealed a role for dynamin 2-mediated exocytosis. Sci. Rep..

[45] Rice C.P.J., Chelakkot V.S., Conohan N.T., Hirasawa K. (2025). Cancer stem cell populations are resistant to 5-aminolevulinic acid-photodynamic therapy (5-ALA-PDT). Sci. Rep..

[46] Chandratre S., Olsen J., Chen B. (2025). A novel acquired resistance mechanism to 5-aminolevulinic acid-mediated photodynamic therapy with ABCG2 inhibition. Photochem. Photobiol..

[47] Liu B., Farrell T.J., Patterson M.S. (2012). Comparison of photodynamic therapy with different excitation wavelengths using a dynamic model of aminolevulinic acid-photodynamic therapy of human skin. J. Biomed. Opt..

[48] Hartl B.A., Hirschberg H., Marcu L., Cherry S.R. (2015). Characterizing low fluence thresholds for in vitro photodynamic therapy. Biomed. Opt. Express.

[49] Tanaka Y., Murayama Y., Matsumoto T., Kubo H., Harada K., Matsuo H., Kubota T., Okamoto K., Otsuji E. (2020). Efficacy of 5-aminolevulinic acid-mediated photodynamic therapy in a mouse model of esophageal cancer. Oncol. Lett..

[50] Howley R., Chandratre S., Chen B. (2023). 5-Aminolevulinic Acid as a Theranostic Agent for Tumor Fluorescence Imaging and Photodynamic Therapy. Bioengineering.

[51] Casas A. (2020). Clinical uses of 5-aminolaevulinic acid in photodynamic treatment and photodetection of cancer: A review. Cancer Lett..

[52] Matsuoka T., Igarashi A., Yamasaki T., Kawakita M. (2026). 5-Aminolevulinic Acid-Based Fluorescence Guidance in Urologic Oncology: Current Status, Pitfalls, and Future Directions. Life.

[53] Gautheron A., Bernstock J.D., Picart T., Guyotat J., Valdés P.A., Montcel B. (2024). 5-ALA induced PpIX fluorescence spectroscopy in neurosurgery: A review. Front. Neurosci..

[54] Lou L., Zhou S., Tan S., Xiang M., Wang W., Yuan C., Gao L., Xiao Q. (2023). Amplifying the efficacy of ALA-based prodrugs for photodynamic therapy using nanotechnology. Front. Pharmacol..

[55] Bhattacharya S., Prajapati B.G., Singh S., Anjum M.M. (2023). Nanoparticles drug delivery for 5-aminolevulinic acid (5-ALA) in photodynamic therapy (PDT) for multiple cancer treatment: A critical review on biosynthesis, detection, and therapeutic applications. J. Cancer Res. Clin. Oncol..

[56] Ebrahimi S., Khaleghi Ghadiri M., Stummer W., Gorji A. (2024). Enhancing 5-ALA-PDT efficacy against resistant tumor cells: Strategies and advances. Life Sci..

[57] Wu R.W., Chu E.S., Yow C.M., Chen J.Y. (2006). Photodynamic effects on nasopharyngeal carcinoma (NPC) cells with 5-aminolevulinic acid or its hexyl ester. Cancer Lett..

[58] Yoon J.H., Yoon H.E., Kim O., Kim S.K., Ahn S.G., Kang K.W. (2012). The enhanced anti-cancer effect of hexenyl ester of 5-aminolaevulinic acid photodynamic therapy in adriamycin-resistant compared to non-resistant breast cancer cells. Lasers Surg. Med..

[59] Teper E., Makhov P., Golovine K., Canter D.J., Myers C.B., Kutikov A., Sterious S.N., Uzzo R.G., Kolenko V.M. (2012). The effect of 5-aminolevulinic acid and its derivatives on protoporphyrin IX accumulation and apoptotic cell death in castrate-resistant prostate cancer cells. Urology.

[60] Feuerstein T., Berkovitch-Luria G., Nudelman A., Rephaeli A., Malik Z. (2011). Modulating ALA-PDT efficacy of mutlidrug resistant MCF-7 breast cancer cells using ALA prodrug. Photochem. Photobiol. Sci..

[61] Shinohara Y., Endo Y., Abe C., Shiba I., Ishizuka M., Tanaka T., Yonemura Y., Ogura S.I., Tominaga M., Yamada H. (2017). Development of a novel Schiff base derivative for enhancing the anticancer potential of 5-aminolevulinic acid-based photodynamic therapy. Photodiagnosis Photodyn. Ther..

[62] Li K., Dong W., Qiu L., Liu Q., Lv G., Peng Y., Xie M., Lin J. (2019). A new GSH-responsive prodrug of 5-aminolevulinic acid for photodiagnosis and photodynamic therapy of tumors. Eur. J. Med. Chem..

[63] Wakui M., Yokoyama Y., Wang H., Shigeto T., Futagami M., Mizunuma H. (2010). Efficacy of a methyl ester of 5-aminolevulinic acid in photodynamic therapy for ovarian cancers. J. Cancer Res. Clin. Oncol..

[64] Iturrioz-Rodríguez N., Bertorelli R., Ciofani G. (2020). Lipid-Based Nanocarriers for The Treatment of Glioblastoma. Adv. Nanobiomed. Res..

[65] Zhang C., Zhao X., Li D., Ji F., Dong A., Chen X., Zhang J., Wang X., Zhao Y., Chen X. (2022). Advances in 5-aminoketovaleric acid(5-ALA) nanoparticle delivery system based on cancer photodynamic therapy. J. Drug Deliv. Sci. Technol..

[66] Brain Gliolan (5-ALA). https://fimedica.pl/gliolan-5-ala/.

[67] Chung C.W., Chung K.D., Jeong Y.I., Kang D.H. (2013). 5-aminolevulinic acid-incorporated nanoparticles of methoxy poly(ethylene glycol)-chitosan copolymer for photodynamic therapy. Int. J. Nanomed..

[68] Yan X., Al-Hayek S., Huang H., Zhu Z., Zhu W., Guo H. (2013). Photodynamic effect of 5-aminolevulinic acid-loaded nanoparticles on bladder cancer cells: A preliminary investigation. Scand. J./Urol..

[69] Yang Y., Hu Y., Du H., Ren L., Wang H. (2018). Colloidal plasmonic gold nanoparticles and gold nanorings: Shape-dependent generation of singlet oxygen and their performance in enhanced photodynamic cancer therapy. Int. J. Nanomed..

[70] Yang K., Yue L., Yu G., Rao L., Tian R., Wei J., Yang Z., Sun C., Zhang X., Xu M. (2021). A hypoxia responsive nanoassembly for tumor specific oxygenation and enhanced sonodynamic therapy. Biomaterials.

[71] Ma X., Qu Q., Zhao Y. (2015). Targeted delivery of 5-aminolevulinic acid by multifunctional hollow mesoporous silica nanoparticles for photodynamic skin cancer therapy. ACS Appl. Mater. Interfaces.

[72] Cao Y., Wu C., Liu Y., Hu L., Shang W., Gao Z., Xia N. (2020). Folate functionalized pH-sensitive photothermal therapy traceable hollow mesoporous silica nanoparticles as a targeted drug carrier to improve the antitumor effect of doxorubicin in the hepatoma cell line SMMC-7721. Drug Deliv..

[73] Niu S., Zhang X., Williams G.R., Wu J., Gao F., Fu Z., Chen X., Lu S., Zhu L.M. (2021). Hollow Mesoporous Silica Nanoparticles Gated by Chitosan-Copper Sulfide Composites as Theranostic Agents for the Treatment of Breast Cancer. Acta Biomater..

[74] Chen Z., Liu W., Liu K., Wang S., Li C., Wu F., Wang S., Tang Y. (2024). Double-layer hollow mesoporous silica nanoparticles for ultrasound-guided photodynamic treatment. Biomed. Mater..

[75] Prieto-Montero R., Arbeloa T., Martínez-Martínez V. (2023). Photosensitizer-Mesoporous Silica Nanoparticles Combination for Enhanced Photodynamic Therapy^†^. Photochem. Photobiol..

[76] Wang C., Li Q., Xiao J., Liu Y. (2023). Nanomedicine-based combination therapies for overcoming temozolomide resistance in glioblastomas. Cancer Biol. Med..

[77] Pedrosa L., Bedia C., Diao D., Mosteiro A., Ferrés A., Stanzani E., Martínez-Soler F., Tortosa A., Pineda E., Aldecoa I. (2023). Preclinical Studies with Glioblastoma Brain Organoid Co-Cultures Show Efficient 5-ALA Photodynamic Therapy. Cells.

[78] Tetard M.C., Vermandel M., Leroy H.A., Leroux B., Maurage C.A., Lejeune J.P., Mordon S., Reyns N. (2016). Interstitial 5-ALA photodynamic therapy and glioblastoma: Preclinical model development and preliminary results. Photodiagnosis Photodyn. Ther..

[79] Vermandel M., Dupont C., Lecomte F., Leroy H.A., Tuleasca C., Mordon S., Hadjipanayis C.G., Reyns N. (2021). Standardized intraoperative 5-ALA photodynamic therapy for newly diagnosed glioblastoma patients: A preliminary analysis of the INDYGO clinical trial. J. Neurooncol..

[80] Briel-Pump A., Beez T., Ebbert L., Remke M., Weinhold S., Sabel M.C., Sorg R.V. (2018). Accumulation of protoporphyrin IX in medulloblastoma cell lines and sensitivity to subsequent photodynamic treatment. J. Photochem. Photobiol. B.

[81] El-Khatib M., Tepe C., Senger B., Dibué-Adjei M., Riemenschneider M.J., Stummer W., Steiger H.J., Cornelius J.F. (2015). Aminolevulinic acid-mediated photodynamic therapy of human meningioma: An in vitro study on primary cell lines. Int. J. Mol. Sci..

[82] Reid P.A., Wilson P., Li Y., Marcu L.G., Bezak E. (2017). Current understanding of cancer stem cells: Review of their radiobiology and role in head and neck cancers. Head Neck.

[83] Liu X.Y., Guo Q.Y., Wang Q., Xu S., Cheng Z., Zhang L., Wang Y.T., Guo X., Liu X.D., Li W.W. (2025). Monitoring Role of Non-invasive Examinations on the Clinical Efficacy of Photodynamic Therapy for Oral Potentially Malignant Disorders. Chin. J. Dent. Res..

[84] Ahn P.H., Quon H., O’Malley B.W., Weinstein G., Chalian A., Malloy K., Atkins J.H., Sollecito T., Greenberg M., McNulty S. (2016). Toxicities and early outcomes in a phase 1 trial of photodynamic therapy for premalignant and early stage head and neck tumors. Oral Oncol..

[85] Ahn P.H., Finlay J.C., Gallagher-Colombo S.M., Quon H., O’Malley BWJr Weinstein G.S., Chalian A., Malloy K., Sollecito T., Greenberg M., Simone CB2nd McNulty S. (2018). Lesion oxygenation associates with clinical outcomes in premalignant and early stage head and neck tumors treated on a phase 1 trial of photodynamic therapy. Photodiagnosis Photodyn. Ther..

[86] Gradishar W.J., Moran M.S., Abraham J., Abramson V., Aft R., Agnese D., Allison K.H., Anderson B., Bailey J., Burstein H.J. (2024). Breast Cancer, Version 3.2024, NCCN Clinical Practice Guidelines in Oncology. J. Natl. Compr. Cancer Netw..

[87] Barzaman K., Karami J., Zarei Z., Hosseinzadeh A., Kazemi M.H., Moradi-Kalbolandi S., Safari E., Farahmand L. (2020). Breast cancer: Biology, biomarkers, and treatments. Int. Immunopharmacol..

[88] Guney Eskiler G., Deveci Ozkan A., Sozen Kucukkara E., Kamanlı A.F., Gunoğlu B., Yıldız M.Z. (2020). Optimization of 5-aminolevulinic acid-based photodynamic therapy protocol for breast cancer cells. Photodiagnosis Photodyn. Ther..

[89] Kamanlı A.F., Yıldız M.Z., Özyol E., Deveci Ozkan A., Sozen Kucukkara E., Guney Eskiler G. (2021). Investigation of LED-based photodynamic therapy efficiency on breast cancer cells. Lasers Med. Sci..

[90] Zhu B., Gu H., Mao Z., Beeraka N.M., Zhao X., Anand M.P., Zheng Y., Zhao R., Li S., Manogaran P. (2024). Global burden of gynaecological cancers in 2022 and projections to 2050. J. Glob. Health.

[91] Tse K.Y., Domingo E.J., Konar H., Kumarasamy S., Pariyar J., Tjokroprawiro B.A., Ushijima K., Inthasorn P., Tan A.L., Wilailak S. (2021). Oncology Committee, Asia and Oceania Federation of Obstetrics and Gynecology. COVID-19 and gynecological cancers: Asia and Oceania Federation of Obstetrics and Gynecology oncology committee opinion. J. Obstet. Gynaecol. Res..

[92] Hu Y., Li Y., Xu Y., Teng Y., Chen J., Ma L. (2022). Topical 5-aminolevulinic acid photodynamic therapy for cervical high-grade squamous intraepithelial lesions. Photodiagnosis Photodyn. Ther..

[93] He G.F., Bian M.L., Zhao Y.W., Xiang Q., Li H.Y., Xiao C. (2008). Apoptosis-inducing effect of 5-aminolevulinic acid-mediated photodynamic therapy (5-ALA-PDT) on cervical cancer cell lines. Ai Zheng.

[94] Han Q., Guo H., Wu Z., Shi J., Zhang X. (2024). Efficacy and Safety of 5-Aminolevulinic Acid Photodynamic Therapy for Treating Cervical and Vaginal Intraepithelial Neoplasia. Pharmaceutics.

[95] Zhao S., Liu D., Shi W., Kang Y., Li Q., Liu Q., Chen M., Li F., Su J., Zhang Y. (2020). Efficacy of a New Therapeutic Option for Vulvar Intraepithelial Neoplasia: Superficial Shaving Combined With Photodynamic Therapy. Lasers Surg. Med..

[96] Teshigawara T., Mizuno M., Ishii T., Kitajima Y., Utsumi F., Sakata J., Kajiyama H., Shibata K., Ishizuka M., Kikkawa F. (2018). Novel potential photodynamic therapy strategy using 5-Aminolevulinic acid for ovarian clear-cell carcinoma. Photodiagnosis Photodyn. Ther..

[97] Schulz W.A., Sørensen K.D. (2019). Epigenetics of Urological Cancers. Int. J. Mol. Sci..

[98] Siegel R.L., Miller K.D., Fuchs H.E., Jemal A. (2022). Cancer statistics, 2022. CA Cancer J. Clin..

[99] Kriegmair M., Baumgartner R., Lumper W., Waidelich R., Hofstetter A. (1996). Early clinical experience with 5-aminolevulinic acid for the photodynamic therapy of superficial bladder cancer. Br. J. Urol..

[100] Waidelich R., Hofstetter A., Stepp H., Baumgartner R., Weninger E., Kriegmair M. (1998). Early clinical experience with 5-aminolevulinic acid for the photodynamic therapy of upper tract urothelial tumors. J. Urol..

[101] Panetta J.V., Cvetkovic D., Chen X., Chen L., Ma C.C. (2020). Radiodynamic therapy using 15-MV radiation combined with 5-aminolevulinic acid and carbamide peroxide for prostate cancer in vivo. Phys. Med. Biol..

[102] Higashi T., Kurokawa Y. (2024). Incidence, mortality, survival, and treatment statistics of cancers in digestive organs-Japanese cancer statistics 2024. Ann. Gastroenterol. Surg..

[103] Kohoutova D., Haidry R., Banks M., Butt M.A., Dunn J., Thorpe S., Lovat L. (2018). Long-term outcomes of the randomized controlled trial comparing 5-aminolaevulinic acid and Photofrin photodynamic therapy for Barrett’s oesophagus related neoplasia. Scand. J. Gastroenterol..

[104] Hino H., Murayama Y., Nakanishi M., Inoue K., Nakajima M., Otsuji E. (2013). 5-Aminolevulinic acid-mediated photodynamic therapy using light-emitting diodes of different wavelengths in a mouse model of peritoneally disseminated gastric cancer. J. Surg. Res..

[105] Hatakeyama T., Murayama Y., Komatsu S., Shiozaki A., Kuriu Y., Ikoma H., Nakanishi M., Ichikawa D., Fujiwara H., Okamoto K. (2013). Efficacy of 5-aminolevulinic acid-mediated photodynamic therapy using light-emitting diodes in human colon cancer cells. Oncol. Rep..

[106] Kumar A., Pecquenard F., Baydoun M., Quilbé A., Moralès O., Leroux B., Aoudjehane L., Conti F., Boleslawski E., Delhem N. (2023). An Efficient 5-Aminolevulinic Acid Photodynamic Therapy Treatment for Human Hepatocellular Carcinoma. Int. J. Mol. Sci..

[107] Ozten O., Guney Eskiler G., Sonmez F., Yıldız M.Z. (2022). Investigation of the therapeutic effect of 5-aminolevulinic acid based photodynamic therapy on hepatocellular carcinoma. Lasers Med. Sci..

[108] Regula J., Ravi B., Bedwell J., MacRobert A.J., Bown S.G. (1994). Photodynamic therapy using 5-aminolaevulinic acid for experimental pancreatic cancer--prolonged animal survival. Br. J. Cancer.

[109] Li L., Yang Y., Yang Z., Zheng M., Luo G., He W., Yin R. (2022). Effects of ALA-PDT on the macrophages in wound healing and its related mechanisms in vivo and in vitro. Photodiagnosis Photodyn. Ther..

[110] Jiang Y., Luo J., Sun K., Li L., Huang X., Chen N., Liu H., Chen J., Lei X. (2023). ALA-PDT shortens the course of antibiotic therapy for skin infection caused by Mycobacterium marinum. Photodiagnosis Photodyn. Ther..

[111] Zhang L., Zeng Q., Li J., Chen N., Tang H., Lei X., Wu J., Cheng Q. (2022). ALA-PDT combined with oral acitretin in the treatment of refractory condyloma acuminatum in anal canal. Photodiagnosis Photodyn. Ther..

[112] Liu H., Wei J., Zhong M., Xu M., Feng S., Peng X., Liu H., Li J., Song W., Zhong Y. (2023). Evaluation of HPV infection helps to direct ALA-PDT of condyloma acuminata. Photodiagnosis Photodyn. Ther..

[113] Chen J., Yuan F., Zheng L., Wen L., Gao M., Zhou W., Fan X. (2023). Limitations of ALA-PDT as a reliable therapy for AK in clinical practice. Photodiagnosis Photodyn. Ther..

[114] Cohen D.K., Lee P.K. (2016). Photodynamic Therapy for Non-Melanoma Skin Cancers. Cancers.

[115] Wang P., Wang B., Zhang L., Liu X., Shi L., Kang X., Lei X., Chen K., Chen Z., Li C. (2023). Clinical practice Guidelines for 5-Aminolevulinic acid photodynamic therapy for acne vulgaris in China. Photodiagnosis Photodyn. Ther..

[116] Bao N., Gu T., Zeng J., Wu Y., Sun Y., Gao X., Chen H. (2022). Combined therapy of 5-aminolevulinic acid photodynamic therapy and intense pulsed light for rosacea. Lasers Med. Sci..

[117] Yao Y., Zuo J., Chen L. (2021). Clinical efficacy of tanshinone capsules combined with varying concentrations of 5-ALA-PDT in the treatment of cystic acne. Am. J. Transl. Res..

[118] Reshetylo S., Narla S., Bakker C., Freeman T., Farah R.S., Hamzavi I.H., Goldfarb N. (2023). Systematic review of photodynamic therapy for the treatment of hidradenitis suppurativa. Photodermatol. Photoimmunol. Photomed..

[119] Zhang F., Li D., Shi L., Gu Y., Xu Y., Wu C. (2021). Efficacy of 5-Aminolevulinic Acid (ALA)-Photodynamic Therapy (PDT) in Refractory Vulvar Lichen Sclerosus: Preliminary Results. Med. Sci. Monit..

[120] Morton C.A., Szeimies R.M., Basset-Seguin N., Calzavara-Pinton P., Gilaberte Y., Haedersdal M., Hofbauer G.F.L., Hunger R.E., Karrer S., Piaserico S. (2019). European Dermatology Forum guidelines on topical photodynamic therapy 2019 Part 1: Treatment delivery and established indications—Actinic keratoses, Bowen’s disease and basal cell carcinomas. J. Eur. Acad. Dermatol. Venereol..

[121] Khan N.H., Mir M., Qian L., Baloch M., Ali Khan M.F., Rehman A.U., Ngowi E.E., Wu D.D., Ji X.Y. (2021). Skin cancer biology and barriers to treatment: Recent applications of polymeric micro/nanostructures. J. Adv. Res..

[122] Ayala E.T.P., Carvalho I.S.E., Antunes C.A., Mahmood A., Requena M.B., Alves F., Pires L., Yakovlev V., Bagnato V.S., Pratavieira S. (2025). Comparative analysis of ALA mediated sonodynamic therapy considering tumor size, light combination and ultrasound delivery in murine cutaneous melanoma. Sci. Rep..

[123] Wang X.L., Wang H.W., Guo M.X., Xu S.Z. (2008). Treatment of skin cancer and pre-cancer using topical ALA-PDT--a single hospital experience. Photodiagnosis Photodyn. Ther..

[124] Córdoba F., Braathen L.R., Weissenberger J., Vallan C., Kato M., Nakashima I., Weis J., von Felbert V. (2005). 5-aminolaevulinic acid photodynamic therapy in a transgenic mouse model of skin melanoma. Exp. Dermatol..

[125] Stummer W., Müther M., Spille D. (2024). Beyond fluorescence-guided resection: 5-ALA-based glioblastoma therapies. Acta Neurochir. (Wien).

[126] Valerio J.E., de Jesús Aguirre Vera G., Zumaeta J., Rea N.S., Fernandez Gomez M.P., Mantilla-Farfan P., Valente L., Alvarez-Pinzon A.M. (2025). Comparative Analysis of 5-ALA and Fluorescent Techniques in High-Grade Glioma Treatment. Biomedicines.

[127] Kurabayashi A., Fukuhara H., Furihata K., Iwashita W., Furihata M., Inoue K. (2024). Photodynamic Diagnosis and Therapy in Non-Muscle-Invasive Bladder Cancer. Cancers.

[128] Zeitouni N.C., Schlesinger T., Kheterpal M., Jolly P.S., Jagdeo J. (2025). 5-aminolevulinic acid photodynamic therapy for the treatment of basal and squamous cell carcinoma: A systematic review. Photodiagnosis Photodyn. Ther..

[129] Sanai N., Tovmasyan A., Tien A.C., Chang Y.W., Margaryan T., Knight W., Hendrickson K., Eschbacher J., Harmon J., Hong A. (2025). An early clinical trial of 5-ALA sonodynamic therapy in recurrent high-grade glioma. Sci. Transl. Med..

[130] Aebisher D., Mytych W., Łoś A., Dynarowicz K., Myśliwiec A., Bartusik-Aebisher D. (2024). The assessment of 5-aminolevulinic acid photodynamic therapy in glioblastomas. Oncologie.

[131] Hwang E., Yun M., Jung H.S. (2023). Mitochondria-targeted organic sonodynamic therapy agents: Concept, benefits, and future directions. Front. Chem..

[132] Gao Z., Zheng J., Yang B., Wang Z., Fan H., Lv Y., Li H., Jia L., Cao W. (2013). Sonodynamic therapy inhibits angiogenesis and tumor growth in a xenograft mouse model. Cancer Lett..

[133] Wang S., Hu Z., Wang X., Gu C., Gao Z., Cao W., Zheng J. (2014). 5-Aminolevulinic acid-mediated sonodynamic therapy reverses macrophage and dendritic cell passivity in murine melanoma xenografts. Ultrasound Med. Biol..

[134] Bonosi L., Marino S., Benigno U.E., Musso S., Buscemi F., Giardina K., Gerardi R., Brunasso L., Costanzo R., Iacopino D.G. (2023). Sonodynamic therapy and magnetic resonance-guided focused ultrasound: New therapeutic strategy in glioblastoma. J. Neurooncol..

[135] Chung I.W., Eljamel S. (2013). Risk factors for developing oral 5-aminolevulinic acid-induced side effects in patients undergoing fluorescence guided resection. Photodiagnosis Photodyn. Ther..

[136] Nonaka M., Yamamoto M., Yoshino S., Umemura S., Sasaki K., Fukushima T. (2009). Sonodynamic therapy consisting of focused ultrasound and a photosensitizer causes a selective antitumor effect in a rat intracranial glioma model. Anticancer Res..

[137] Song D., Yue W., Li Z., Li J., Zhao J., Zhang N. (2014). Study of the mechanism of sonodynamic therapy in a rat glioma model. Onco Targets Ther..

[138] Suehiro S., Ohnishi T., Yamashita D., Kohno S., Inoue A., Nishikawa M., Ohue S., Tanaka J., Kunieda T. (2018). Enhancement of antitumor activity by using 5-ALA-mediated sonodynamic therapy to induce apoptosis in malignant gliomas: Significance of high-intensity focused ultrasound on 5-ALA-SDT in a mouse glioma model. J. Neurosurg..

[139] Sun Y., Wang H., Wang P., Zhang K., Geng X., Liu Q., Wang X. (2019). Tumor targeting DVDMS-nanoliposomes for an enhanced sonodynamic therapy of gliomas. Biomater. Sci..

[140] Chandratre S., Merenich D., Myers K., Chen B. (2024). 5-Aminolevulinic acid-mediated photodynamic therapy in combination with kinase inhibitor lapatinib enhances glioblastoma cell death. Apoptosis.

[141] Kogias E., Fotakopoulos G., Georgakopoulou V.E., Kagkouras I., Spandidos D.A., Trakas N., Foroglou N. (2026). 5-ALA vs. fluorescein guided resection for high-grade gliomas: A systematic review and meta-analysis. Mol. Clin. Oncol..

[142] Shah H.A., Leskinen S., Khilji H., Narayan V., Ben-Shalom N., D’Amico R.S. (2022). Utility of 5-ALA for fluorescence-guided resection of brain metastases: A systematic review. J. Neurooncol..

[143] Collins V.G., Kanodia C., Yahya Q.B., Liistro M., Kaliaperumal C. (2025). 5-Aminolevulinic acid (5-ALA) in paediatric brain tumour surgery-a systematic review and exploration of fluorophore alternatives. Childs Nerv. Syst..

[144] Wu Z.X., Yang Y., Wang J.Q., Narayanan S., Lei Z.N., Teng Q.X., Zeng L., Chen Z.S. (2021). Overexpression of ABCG2 Confers Resistance to MLN7243, a Ubiquitin-Activating Enzyme (UAE) Inhibitor. Front. Cell Dev. Biol..

[145] Pepper N.B., Troschel F.M., Stummer W., Eich H.T. (2025). 5-Aminolevulinic acid as an emerging radiosensitizer for radiodynamic therapy in solid tumors: A systematic review of available data and clinical potential. Strahlenther. Onkol..

[146] Bordoloi B., Goswami A., Roy D., Goswami P., Das I. (2024). Efficacy of Aminolevulinic Acid Mediated Photodynamic Therapy in the Treatment of Oral Premalignant Lesions: A Systematic Review. Asian Pac. J. Cancer Prev..

[147] Hua Y., Tian X., Zhang X., Song G., Liu Y., Zhao Y., Gao Y., Yin F. (2024). Applications and challenges of photodynamic therapy in the treatment of skin malignancies. Front. Pharmacol..

[148] Bader N., Hajosch A., Peschmann C., Stucke-Straub K., Wirtz C.R., Kast R.E., Halatsch M.E., Capanni F., Karpel-Massler G. (2025). Assessment of Photodynamic Therapy Penetration Depth in a Synthetic Pig Brain Model: A Novel Approach to Simulate the Reach of PDT-Mediated Effects In Vitro. Pharmaceuticals.

[149] Mischkulnig M., Roetzer-Pejrimovsky T., Lötsch-Gojo D., Kastner N., Bruckner K., Prihoda R., Lang A., Martinez-Moreno M., Furtner J., Berghoff A. (2022). Heme Biosynthesis Factors and 5-ALA Induced Fluorescence: Analysis of mRNA and Protein Expression in Fluorescing and Non-fluorescing Gliomas. Front. Med..

[150] Pogue B.W., Chen B., Ochoa M.I., Petusseau A., Liu A., Gibson A.L.F., Maytin E.V., Wilson B.C. (2025). Emerging uses of 5-aminolevulinic-acid-induced protoporphyrin IX in medicine: A review of multifaceted, ubiquitous, molecular diagnostic, therapeutic, and theranostic opportunities. J. Biomed. Opt..

[151] Mansi M., Howley R., Chandratre S., Chen B. (2022). Inhibition of ABCG2 transporter by lapatinib enhances 5-aminolevulinic acid-mediated protoporphyrin IX fluorescence and photodynamic therapy response in human glioma cell lines. Biochem. Pharmacol..

[152] Samatar A.A., Poulikakos P.I. (2014). Targeting RAS-ERK signalling in cancer: Promises and challenges. Nat. Rev. Drug Discov..

[153] Chandratre S., Olsen J., Howley R., Chen B. (2023). Targeting ABCG2 transporter to enhance 5-aminolevulinic acid for tumor visualization and photodynamic therapy. Biochem. Pharmacol..

[154] Qi Q., Gu R., Zhu J., Anderson K.E., Ma X. (2024). Roles of the ABCG2 Transporter in Protoporphyrin IX Distribution and Toxicity. Drug Metab. Dispos..

[155] Howley R., Olsen J., Chen B. (2024). Effectiveness of lapatinib for enhancing 5-aminolevulinic acid-mediated protoporphyrin IX fluorescence and photodynamic therapy in human cancer cell lines with varied ABCG2 activities. Photochem. Photobiol..

[156] Liu Y., Mensah S.K., Farias S., Khan S., Hasan T., Celli J.P. (2024). Efficacy of photodynamic therapy using 5-aminolevulinic acid-induced photosensitization is enhanced in pancreatic cancer cells with acquired drug resistance. Photodiagnosis Photodyn. Ther..

[157] Nakayama T., Otsuka S., Kobayashi T., Okajima H., Matsumoto K., Hagiya Y., Inoue K., Shuin T., Nakajima M., Tanaka T. (2016). Dormant cancer cells accumulate high protoporphyrin IX levels and are sensitive to 5-aminolevulinic acid-based photodynamic therapy. Sci. Rep..

[158] Ruhi M.K. (2025). Protoporphyrin IX Beyond Conventional Applications: A Review of Emerging Research Directions. Life.

[159] Balakirski G., Lehmann P., Szeimies R.M., Hofmann S.C. (2024). Photodynamic therapy in dermatology: Established and new indications. J. Dtsch. Dermatol. Ges..

[160] Jing Y., Shu R., Wu T., Liu D., Luo X., Sun J., Chen F. (2024). Clinical efficacy of photodynamic therapy of oral potentially malignant disorder. Photodiagnosis Photodyn. Ther..

[161] Erk B., Kamanli A.F., Guney Eskiler G. (2024). The therapeutic efficacy of 5-ALA based photodynamic therapy and chemotherapy combination in triple negative breast cancer cells. Lasers Med. Sci..

[162] Figueras J.H., Chan D., Maheshwer B., Erwin J., Thomson C., Dixon T., Grawe B.M., Thompson A.R. (2023). Development of an Orthopedic Surgery Anatomy Curricular Model for Fourth Year Medical Students Using a Modified Delphi Method. J. Surg. Educ..

[163] Laplante M., Sabatini D.M. (2012). mTOR signaling in growth control and disease. Cell.

[164] Tilekar K., Upadhyay N., Iancu C.V., Pokrovsky V., Choe J.Y., Ramaa C.S. (2020). Power of two: Combination of therapeutic approaches involving glucose transporter (GLUT) inhibitors to combat cancer. Biochim. Biophys. Acta Rev. Cancer.

[165] Hu-Lieskovan S., Robert L., Homet Moreno B., Ribas A. (2014). Combining targeted therapy with immunotherapy in BRAF-mutant melanoma: Promise and challenges. J. Clin. Oncol..

[166] Sun C., Fang Y., Yin J., Chen J., Ju Z., Zhang D., Chen X., Vellano C.P., Jeong K.J., Ng P.K. (2017). Rational combination therapy with PARP and MEK inhibitors capitalizes on therapeutic liabilities in *RAS* mutant cancers. Sci. Transl. Med..

[167] Wang L., Chelakkot V.S., Newhook N., Tucker S., Hirasawa K. (2023). Inflammatory cell death induced by 5-aminolevulinic acid-photodynamic therapy initiates anticancer immunity. Front. Oncol..

[168] Han Y., Tian X., Zhai J., Zhang Z. (2024). Clinical application of immunogenic cell death inducers in cancer immunotherapy: Turning cold tumors hot. Front. Cell Dev. Biol..

[169] Sun Z., Zhao M., Wang W., Hong L., Wu Z., Luo G., Lu S., Tang Y., Li J., Wang J. (2023). 5-ALA mediated photodynamic therapy with combined treatment improves anti-tumor efficacy of immunotherapy through boosting immunogenic cell death. Cancer Lett..

[170] Zhao R., Li S., Zhao J., Yao C. (2025). Advancements in Nano-Delivery Systems for Photodynamic and Photothermal Therapy to Induce Immunogenic Cell Death in Tumor Immunotherapy. Int. J. Nanomed..

[171] Maegawa H., Kohashi M., Harada Y., Tanaka A., Kajiwara S., Fujimoto T., Atagi H., Kaneda K. (2025). Antitumor immunostimulatory effect via cell-killing action of a novel extracorporeal blood circulating photodynamic therapy system using 5-aminolevulinic acid. Sci. Rep..

[172] Tanaka M., Kataoka H., Yano S., Sawada T., Akashi H., Inoue M., Suzuki S., Inagaki Y., Hayashi N., Nishie H. (2016). Immunogenic cell death due to a new photodynamic therapy (PDT) with glycoconjugated chlorin (G-chlorin). Oncotarget.

